# Alter Game: A Study Protocol on a Virtual “Serious Game” for Relapse Prevention in Patients With Gambling Disorder

**DOI:** 10.3389/fpsyt.2022.854088

**Published:** 2022-04-01

**Authors:** Rosaria Giordano, Maria Anna Donati, Lorenzo Zamboni, Francesca Fusina, Caterina Primi, Fabio Lugoboni

**Affiliations:** ^1^Department of Internal Medicine, Unit of Addiction Medicine, G.B. Rossi Hospital, Verona, Italy; ^2^Department of Neuroscience, Biomedicine and Movement, University of Verona, Verona, Italy; ^3^Department of Neuroscience, Psychology, Drug, and Child's Health, Section of Psychology, University of Florence, Florence, Italy; ^4^Department of General Psychology, University of Padova, Padua, Italy; ^5^Padova Neuroscience Center, University of Padova, Padua, Italy

**Keywords:** gambling disorder, virtual cue-exposure therapy, serious game, relapse prevention, craving

## Abstract

Cognitive behavioral therapy (CBT) is the most successful protocol in gambling disorder (GD) treatment. However, it presents some weaknesses, especially concerning relapse prevention (RP). RP is one of the most important therapeutic steps, aiming at managing cravings and to avoid future relapse increasing perceived self-efficacy. Encouraging results come from the blending of psychotherapy and virtual reality (VR), containing gambling cues. The goal of *Alter Game* (approved by the Ethical Commission, Prot. No. 69346) is verifying the efficacy of an innovative psychological treatment for GD based on the integration of traditional CBT therapy and an immersive VR cue exposure therapy using a serious virtual game, which is a game designed for purposes other than entertainment. RP in virtual cue-exposure therapy allows pathological gamblers to manage the urge to gamble and to avoid relapse by becoming aware of which internal and external triggers are related to craving. We hypothesize that the integrated intervention will be more effective than simple CBT with regard to self-efficacy, craving, and gambling-related distortions. Four virtual ecological environments were developed, and a virtual app, Exludo, interfaced with a computerized multiparametric acquisition system for biofeedback, was created. A sample of about 60 patients aged between 18 and 65 with GD referring to the Addiction Medicine Unit of Verona (Rossi Hospital) will be recruited. Patients will be randomly assigned to the CBT group (16 CBT sessions) or the CBT + VR group (8 CBT sessions + 8 VR cue-exposure therapy sessions). The MCMI-III, the BIS-11, and the SOGS will be used to evaluate inclusion and exclusion criteria, while the *Gambling Related Cognitions Scale* and the *Multidimensional Gambling Self-Efficacy Scale* will be used to verify changes as a function of the treatment. Craving will be evaluated through VAS, and psychophysiological variables will be assessed through biofeedback. A pre-test/post-test experimental design with a 1-month follow-up will be conducted. This study will examine an innovative psychotherapeutic protocol for GD treatment, and it will help in identifying new virtual tools to increase the efficacy of traditional therapeutic approaches that could also be applied to treat other addictions.

## Introduction

Gambling disorder (GD) is the first behavioral addiction to be approved in the DSM-5 [Diagnostic and Statistical Manual of Mental Disorders, 5th ed. (1)], in which it is included in addictive disorders rather than in impulse-control disorders. Indeed, GD presents more similarities with substance-use disorders than with impulse-control disorders (2, 3), as the persistent and current pattern of gambling associated with GD presents symptoms such as tolerance and withdrawal that are typical of addiction (1). In Europe, GD prevalence rates range between 0.12 and 5.8% (1), but it often goes undiagnosed and untreated, and it is associated with negative health measures (4, 5). GD is one of the *new addictions* that affect people in terms of substantial clinical distress in terms of their social life, work, training, and education (1), as well as from a psychosomatic point of view. Thus, focusing on the efficacy of diagnostic and therapeutic measures for this disorder with a scientific approach is especially relevant to deliver patient-tailored treatments (6). Further studies are needed to compare the efficacy and acceptability of individual and combined psychosocial and pharmacological interventions to deliver patient-tailored treatments.

To address this issue, the current study protocol—*Alter Game*- is aimed at (1) developing an innovative psychological treatment for GD by using a virtual reality (VR) approach, and (2) verifying its efficacy. The rationale for using this approach in GD treatment lies in the fact that cognitive behavioral therapy (CBT) is the most grounded and effective theoretical and applied approach to treating addiction (7). Moreover, a review of evidence-based best practices in GD treatment demonstrated that psychological interventions are the most successful protocols, especially concerning motivational and CBT therapies (6, 8), confirming the findings of the Cochrane review (9). In GD treatment, CBT considers cue exposure therapy (10), cognitive restructuring, problem solving techniques, social skills training, and relapse prevention (11) as fundamental steps. Among them, relapse prevention (RP) is one of the most important steps for GD. Based on the RP model proposed by Marlatt and Gordon (12) for substance abuse, the RP model applied in CBT is focused on a set of cognitive and behavioral strategies to prevent or limit relapse episodes. In general, the RP model refers to immediate determinants (e.g., high-risk situations, coping skills, outcome expectancies, the abstinence violation effect) and covert antecedents (e.g., craving, lifestyle imbalances, and urges) (13). RP therapy aims to find, along with the patients, the factors or situations that can precipitate or contribute to relapse episodes and teaches patients how to manage them, increasing their perceived self-efficacy. Self-efficacy is the degree to which an individual feels confident and capable of performing a certain behavior in a specific situational context (14). The presence of low perceived self-efficacy may contribute to maintaining pathological gambling behavior (15). CBT presents different advantages in pathological gambling treatment; there are lower risks of relapse during the follow-up period, and it has a limited duration and involves structured interventions with long-term positive effects (16, 17). On the other hand, CBT presents some limits. Indeed, difficulties were detected in the treatment of pathological gamblers with high levels of sensation seeking and problematic emotional regulation, in which a higher drop-out rate and higher frequency of relapse emerged (18, 19). Thus, the improvement of CBT efficacy for GD is necessary, especially concerning RP. For this reason, there is a need for innovative methodologies to support traditional intervention techniques.

Encouraging results in this regard have come from the blending of psychotherapy and VR, an immersive computer-generated three-dimensional virtual environment, which is viewed through a head-mounted display (HMD) and interacted with by using controllers (20, 21). VR has been the focus of international interest for the last 20 years or so. Virtual applications are used, in particular, in psychotherapeutic interventions for the treatment of post-traumatic stress disorder (22), social anxiety (23, 24), paranoia (25), specific phobias, such as acrophobia (26), aerophobia (27, 28) and arachnophobia (29, 30). Recent literature has also recently pointed to the usefulness of VR in the treatment of GD (31–34), since VR is a methodological approach that allows to recreate a realistic ecological representation of craving situations (35–37). It has been shown that virtual environments containing gambling cues elicit cravings of the same intensity as those induced by real gaming-related stimuli (e.g., real and virtual slot machines) (31). Craving is defined by the DSM-5 as a persistent, intense or irresistible desire for a specific substance (1) or, in the case of gambling, for an addictive behavior. Craving presents cognitive, emotional, and behavioral characteristics, and it is classified in three different types: reward, relief, and obsessive craving (38); therefore, RP in virtual cue-exposure therapy allows pathological gamblers to manage their urge to gamble and to avoid relapse, given that they become aware of which internal and external triggers are related to craving. The idea of targeting VR for the implementation of RP interventions is supported by other advantages of its application in psychotherapy. Indeed, a patient can experience himself/herself within a protected environment, taking advantage of the possibility of interrupting the virtual experience when he/she feels discomfort or difficulties. The virtual exposure is constantly supervised by the therapist through a monitor: what gamblers see through the HMD is, at the same time, watched by the psychotherapist as well. Furthermore, the ecological validity of the environments guarantees greater generalizability of the skills acquired in VR to daily life environments and situations (39).

However, studies on the use of VR with gamblers present some limits. Some studies were conducted on small samples that, sometimes, did not include pathological gamblers (10, 31, 34). Other important weaknesses that could be improved are the following: (a) distinguishing the physical activation due to gambling craving from the one due to possible cybersickness, i.e., discomfort, apathy, nausea, drowsiness, disorientation, eyestrain, and fatigue that may be elicited by an experience in immersive VR (40, 41); (b) using virtual gambling stimuli in association with other virtual addiction triggers (such as alcohol), (c) using VR with other psychotherapeutic techniques, like mindfulness (31); (d) exploring the role of sense of presence in virtual experiences. Sense of presence is a mental manifestation (42), a cognitive state which is consistent with a subjective feeling of “being there,” in a given virtual environment, which is different from the physical one and whose fruition is mediated by a device (43).

Keeping this background into account, the present *Alter Game* study was designed. *Alter Game* aims at developing an innovative psychological treatment for GD by using VR and verifying the efficacy of an innovative psychological treatment for GD based on the integration of traditional CBT therapy and VR approach. To that goal, we aimed at creating a virtual serious game to prevent relapse in gambling behavior by increasing self-efficacy in managing craving and emotional states and by reducing perceived gambling craving. Indeed, virtual environments are made *ad hoc* for the exposure of patients to specific gambling cues, directed at the detection and management of craving. We chose self-efficacy and craving as dependent variables because they are key aspects in maintaining abstention from gambling. Gamblers that perceive good self-efficacy in managing cravings present a lower probability to relapse, on the basis of the Marlatt RP model (12); thus, craving itself is an important relapse risk factor to be managed and recognized. On the other hand, perceived self-efficacy is a protective factor against relapse (12). Moreover, in response to the above-described limitations in previous studies applying VR approach to gambling treatment, *Alter Game* aims at evaluating the sense of presence through multiple means: the *Independent Television Commission's Sense of Presence Inventory* [ITC-SOPI (44)]; the use of diaphragmatic breathing to manage craving; the presence of cigarette packs, lit cigarettes, snacks, alcoholic beverages (wine, beer), and wine glasses in the specific virtual environments; the use of biofeedback to measure the psychophysiological activation. Cue reactivity, indeed, is measured across several domains of human functioning. The most commonly collected measures are self-reports that assess craving or the desire for a particular substance and physiological responses, which usually include those controlled by the autonomic nervous system, such as heart rate, skin conductance, and skin temperature (45). Psychophysiological arousal is a potential proxy indicator of craving as a reaction to gambling-related stimuli. Conditioned stimuli associated with gambling could simultaneously elicit both subjective craving and psychophysiological arousal (46).

Our hypothesis is that an integrated intervention with VR will be more effective than simple CBT for improving self-efficacy and lowering cravings. Furthermore, in line with CBT, we expect gambling-related cognitive distortions to decrease. This goal is very important because gambling-related cognitive distortions mediate the relationship between depressive symptoms and gambling severity (47) and contribute to the maintenance of maladaptive emotional regulation styles to reinforce and support biased beliefs about gambling outcomes and controllability (48).

## Methods and Analysis

*Alter Game* was conceptualized by the Addiction Medicine Unit's interdisciplinary team at the G.B. Rossi Hospital in Verona (Italy). The hospital has signed a collaboration agreement with the Department of Neuroscience, Psychology, Drug, and Child's Health, Section of Psychology in University of Florence (NEUROFARBA—Italy) for methodological and psychometric consultancy related to the study protocol.

### Study Protocol Steps

#### Development and Creation of Virtual Environments

**Figure 1:**
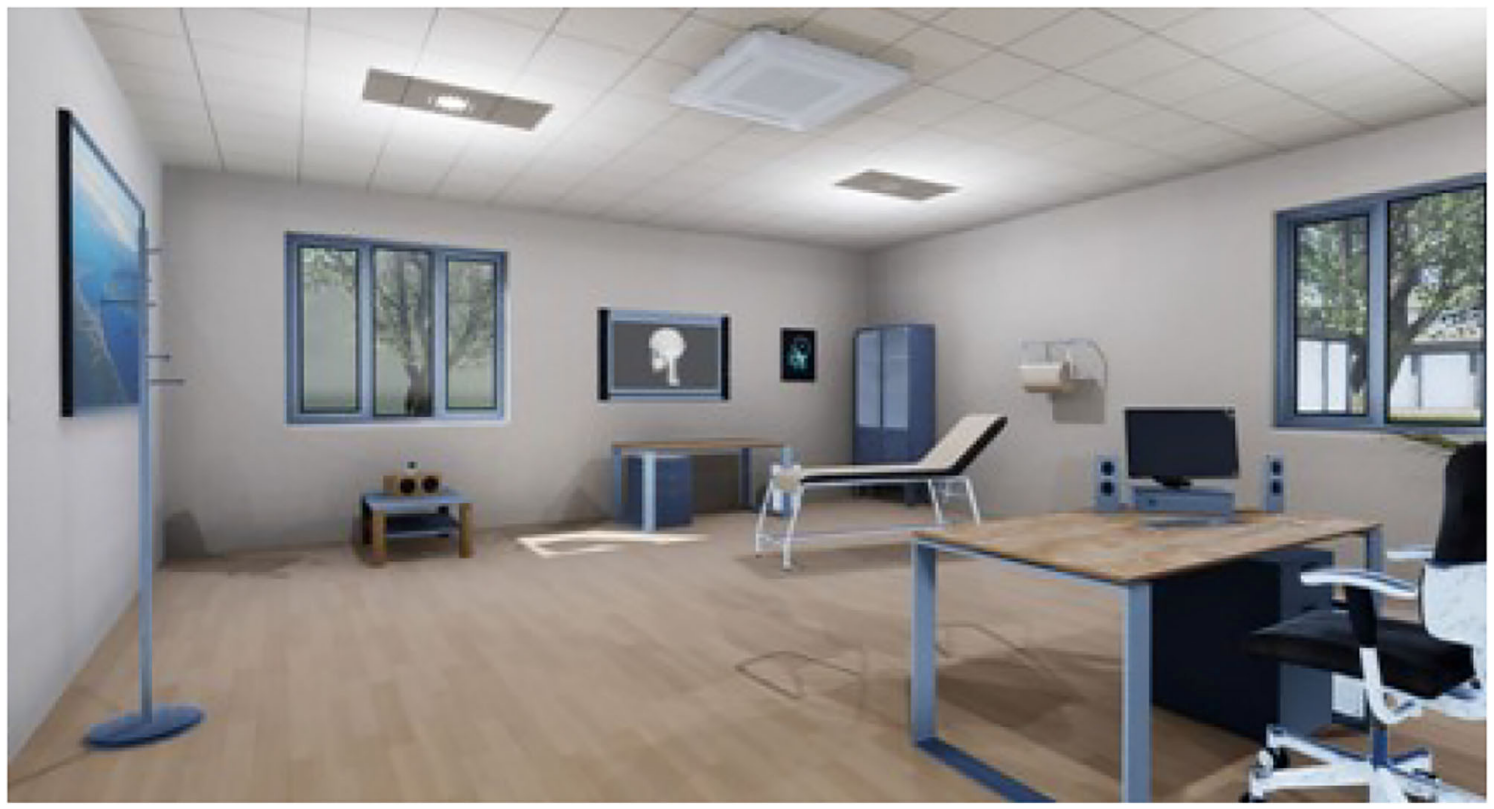
Medical Psychological Office.

**Figure 2:**
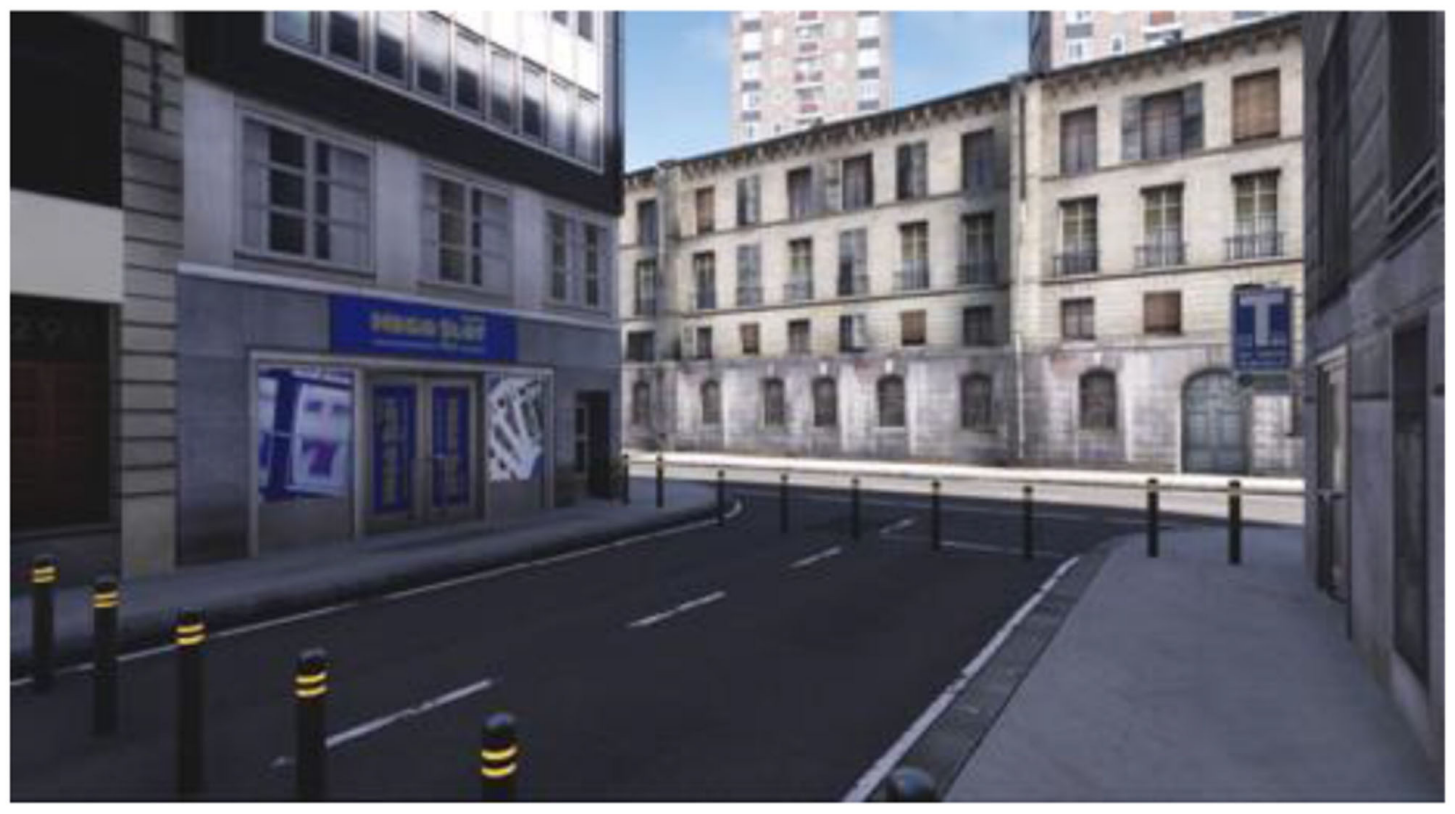
A city street with access to a tobacco store and a slot room.

**Figure 3:**
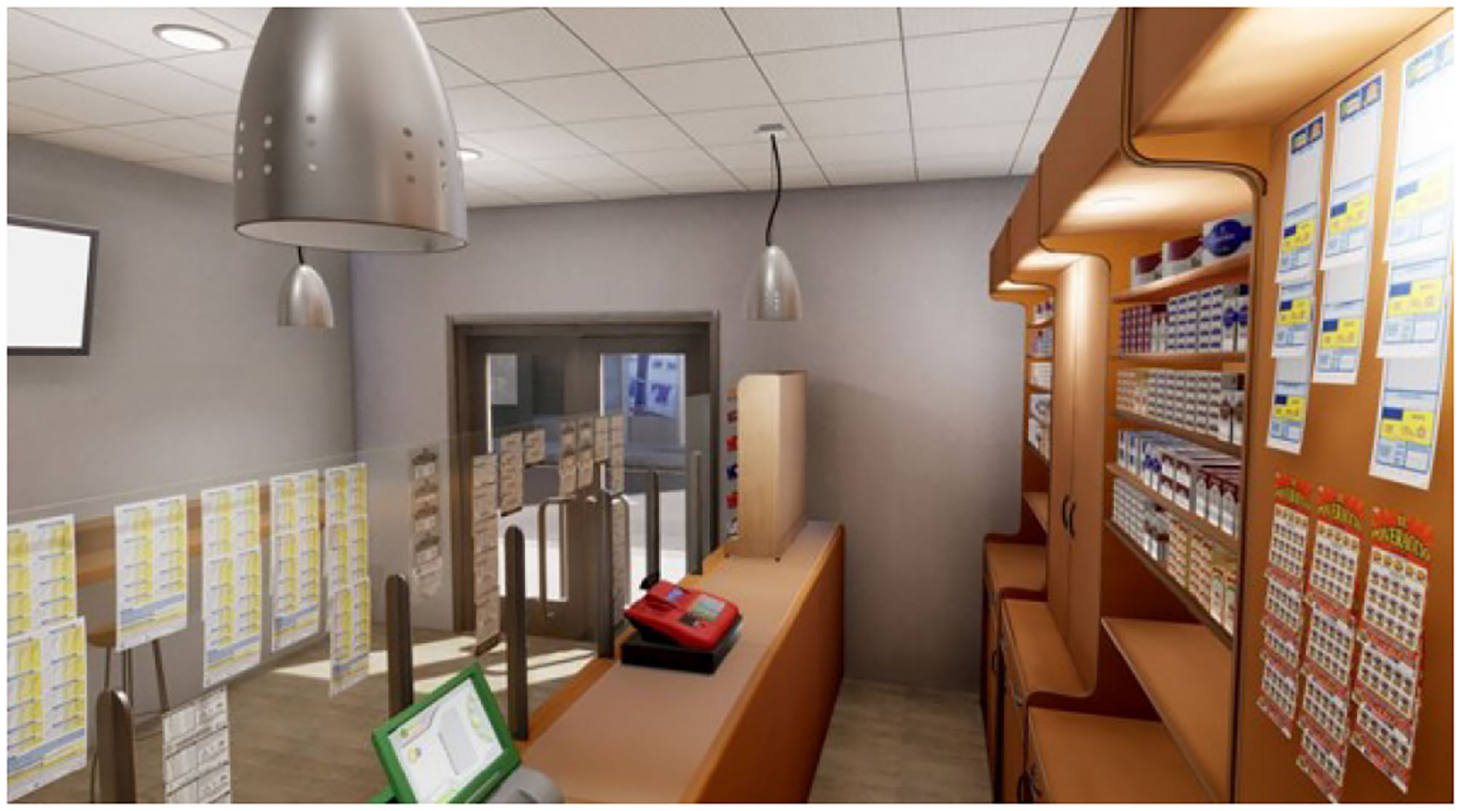
A tobacco store.

**Figure 4:**
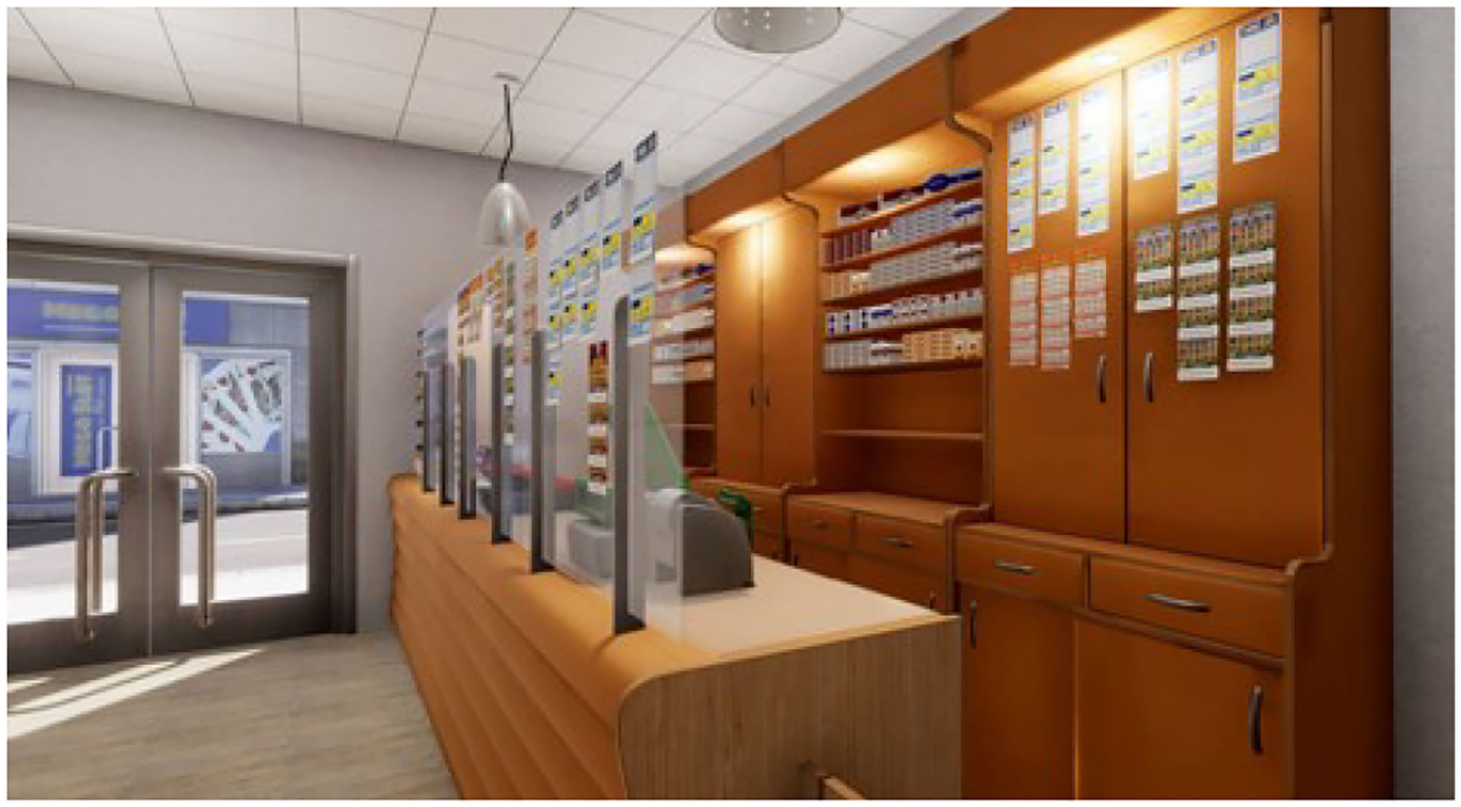
A tobacco store with a view of the slot room.

**Figure 5:**
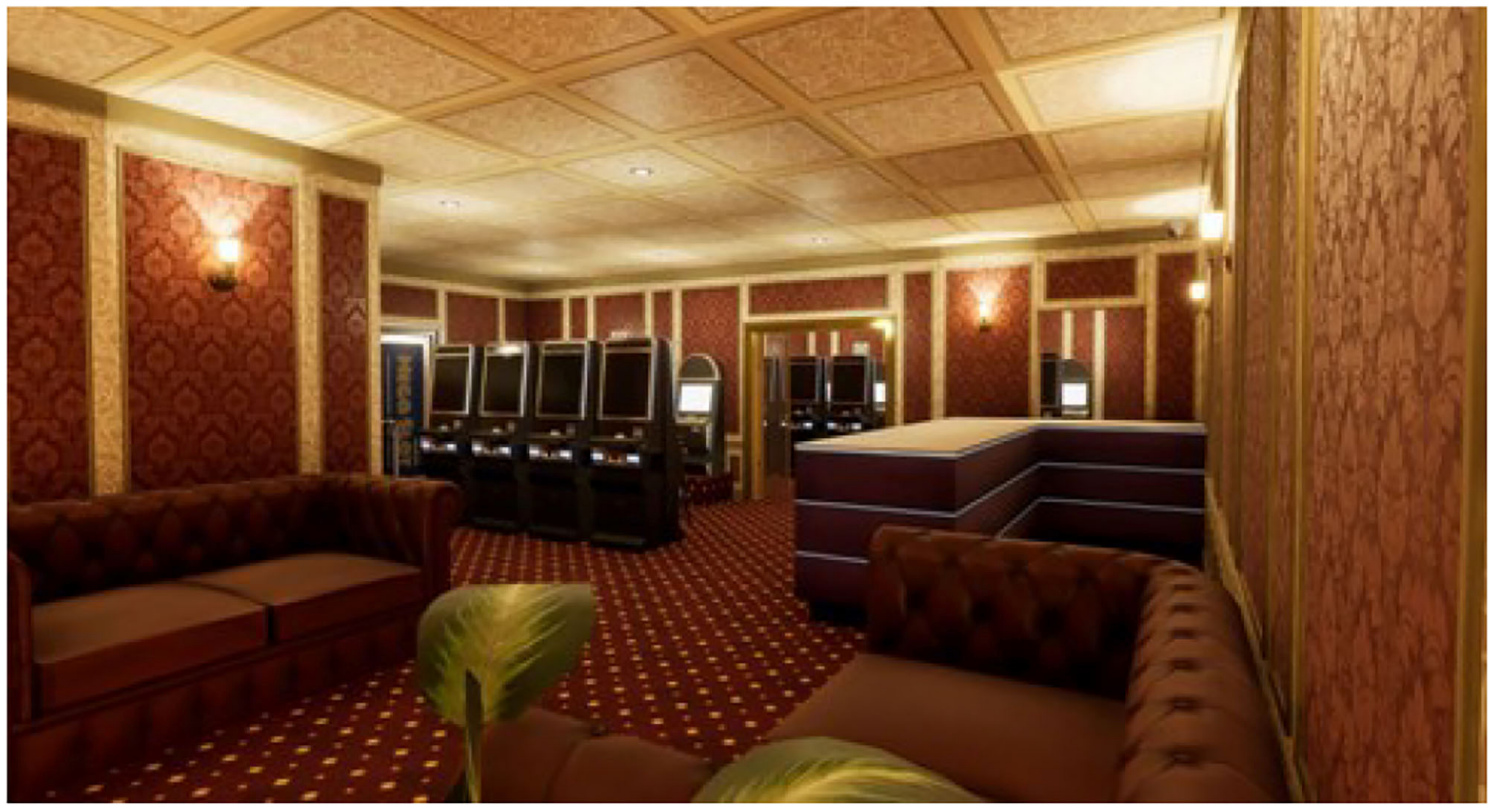
A slot room.

**Figure 6:**
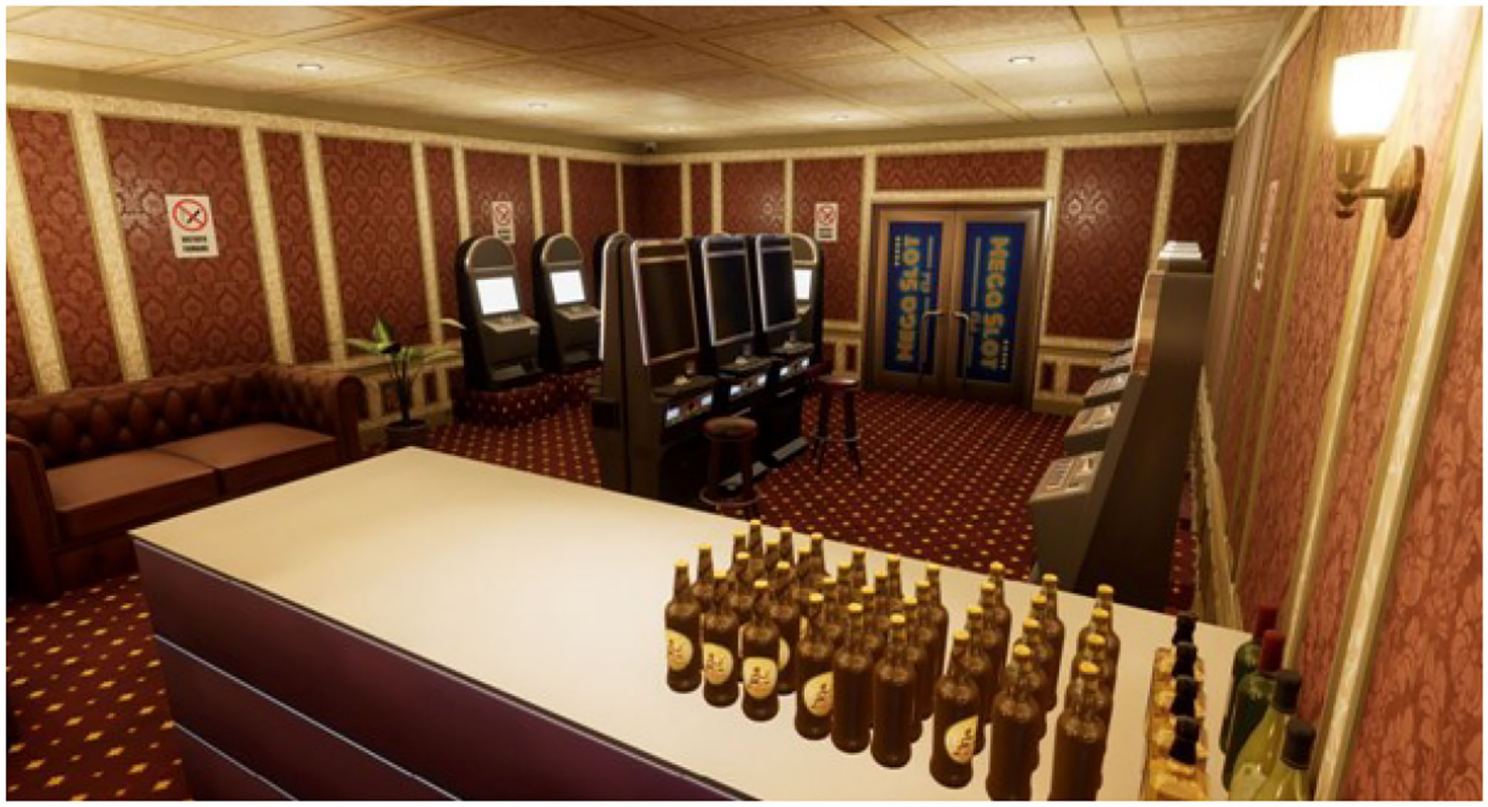
A slot room with some alcohol cues present in Exludo.

**Figure 7:**
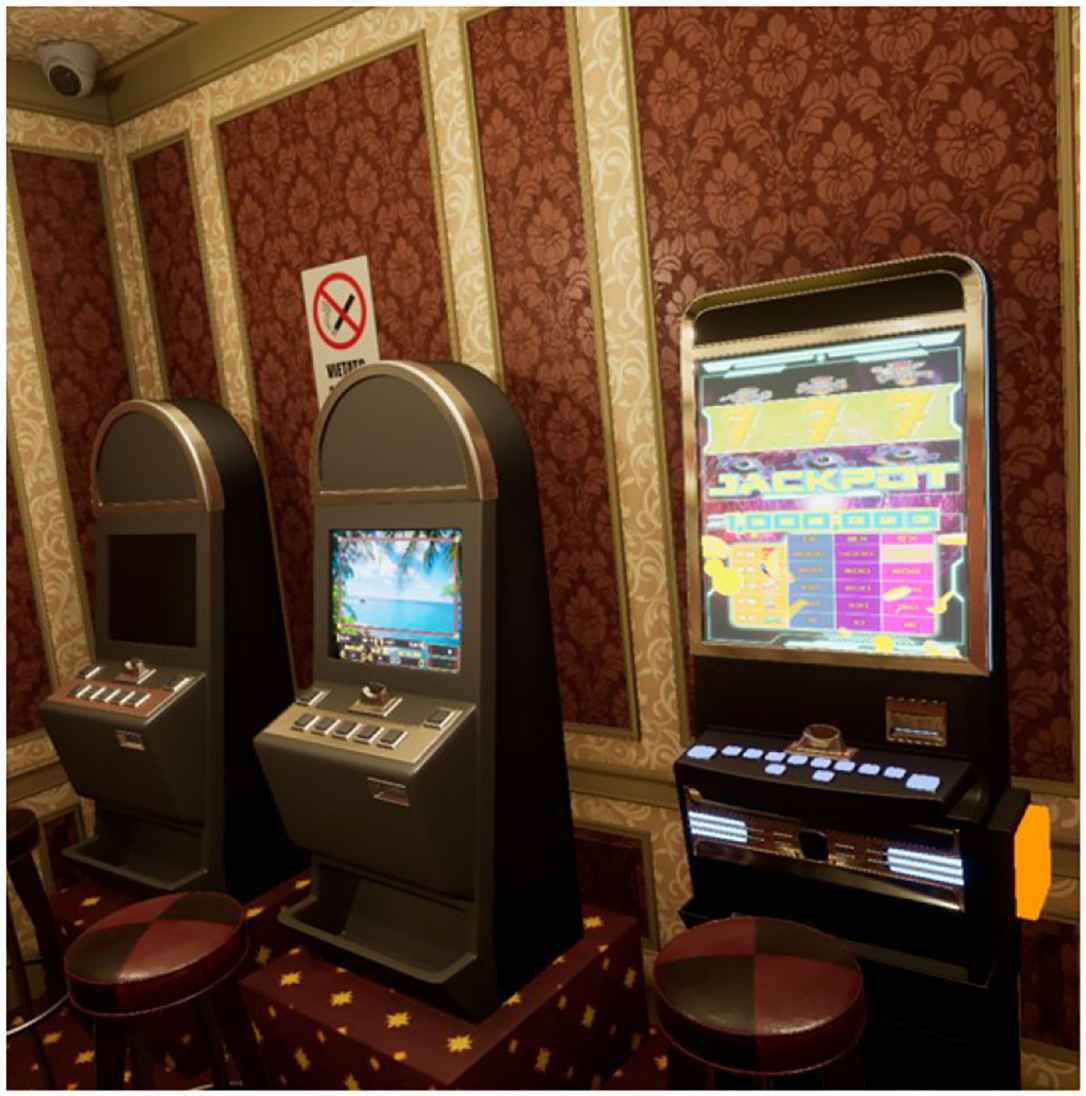
The non-smoking slot room with some slots turned off and on.

**Figure 8:**
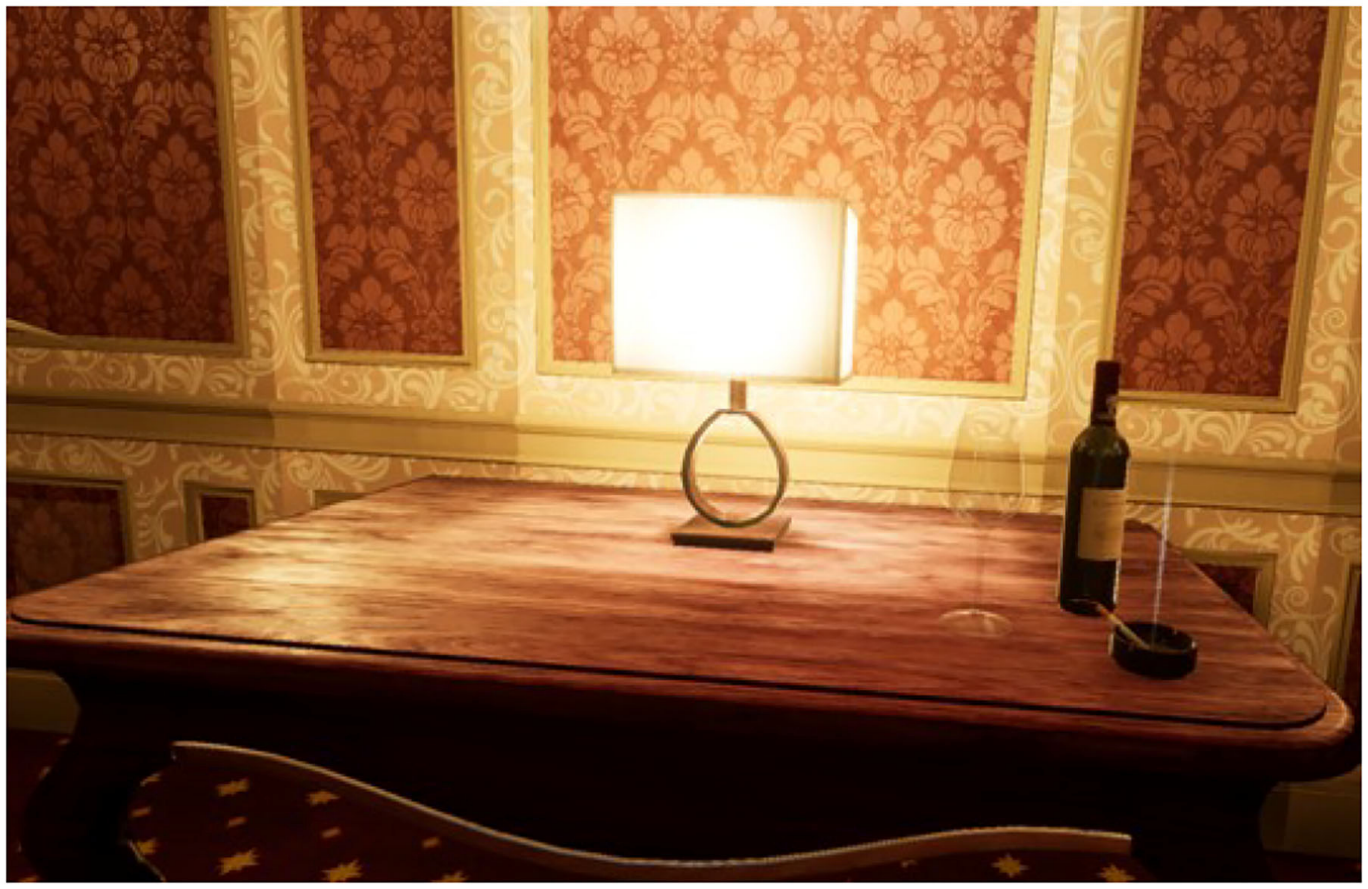
A red wine bottle, a wineglass and a lit cigarette in the slot room.

To carry out the study, four virtual ecological environments structured according to the above goals have been developed, created, and tested. We built a cue-free virtual medical psychological office (see Figure 1), a city street with access to a tobacco store and a slot room (see Figure 2), a tobacco store (see Figures 3, 4), and a slot room (see Figures 5–8). All of the environments, except for the medical psychological office, have also been structured as empty, i.e., without cues, because cue exposure comprises a series of hierarchical stages that gradually provoke craving (10). We chose these environments on the basis of the most frequently reported gambling activities by gamblers (49) and the activities most commonly associated with higher rates of problematic gambling according to literature (50, 51). The medical psychological office was created to facilitate gamblers in the acquisition of skills to use virtual instrumentation and to manage the relaxation phase present in systematic desensitization during the virtual cue-exposure therapy. Gamblers will interact only with a radio to play a relaxing song. They will not be permitted to gamble in any one environments: it will not be possible to start the slot machine and play scratch cards, for example. More emphasis was placed on game-related sound stimuli that constitute a strong appeal for the patient and, therefore, require a targeted desensitization intervention (52, 53). In the slot room, a non-smoking room was created. In the tobacco store, a clock was inserted and marked the exact, real time.

**Figure 9:**
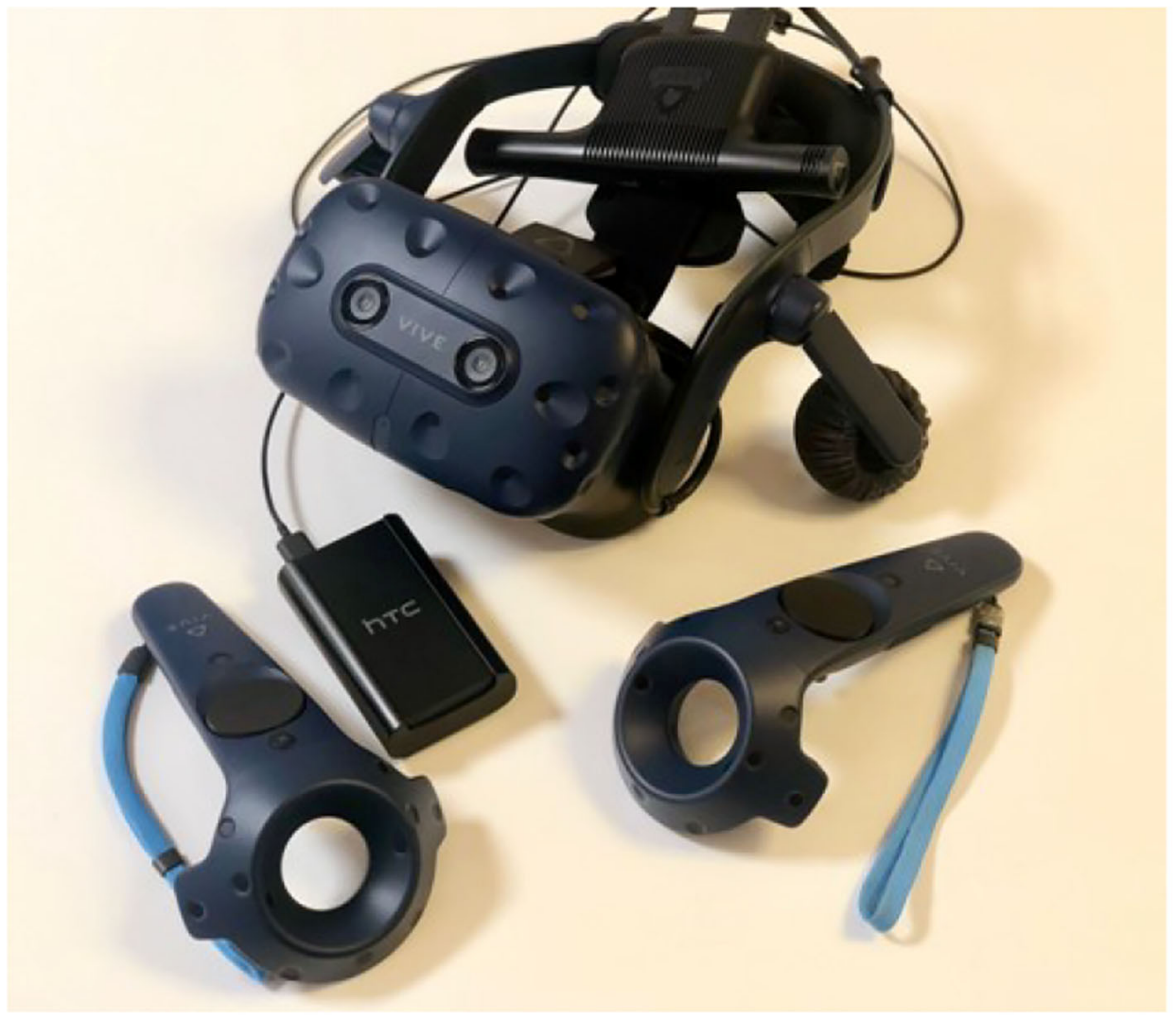
HTC Vive PRO.

Virtual environments have been created in photorealistic quality and in a “cybersickness-free” mode in order to offer the patient a virtual experience that would be as comfortable as possible, without any unpleasant effects. Indeed, cybersickness consists in a feeling of malaise due to headaches, nausea, vomiting, or dizziness, and it is triggered by the incompatibility between the inputs reaching the brain through the visual sensory channel and those responding to one's real movement (54). To achieve this goal, patients will be able to move within the virtual environment through “real” steps whose movement would be faithfully reproduced in the virtual environment. Moreover, gamblers may use teleportation if the position to be reached is distant and beyond the play area. Through the joystick, it is possible to point to the place to reach, and the virtual experience will restart exactly from the selected point. VR environments run on this VR hardware requirement: (a) HTC Vive PRO Full Kit with a wireless adapter (see Figure 9); (b) PC-Gaming Intel Core i7-9700K—GeForce RTX 2070 8GB-−16GB DDR4-−480GB SSD—Windows 10—Wi-Fi; (c) a 49“ or 55” TV monitor.

**Figure 10:**
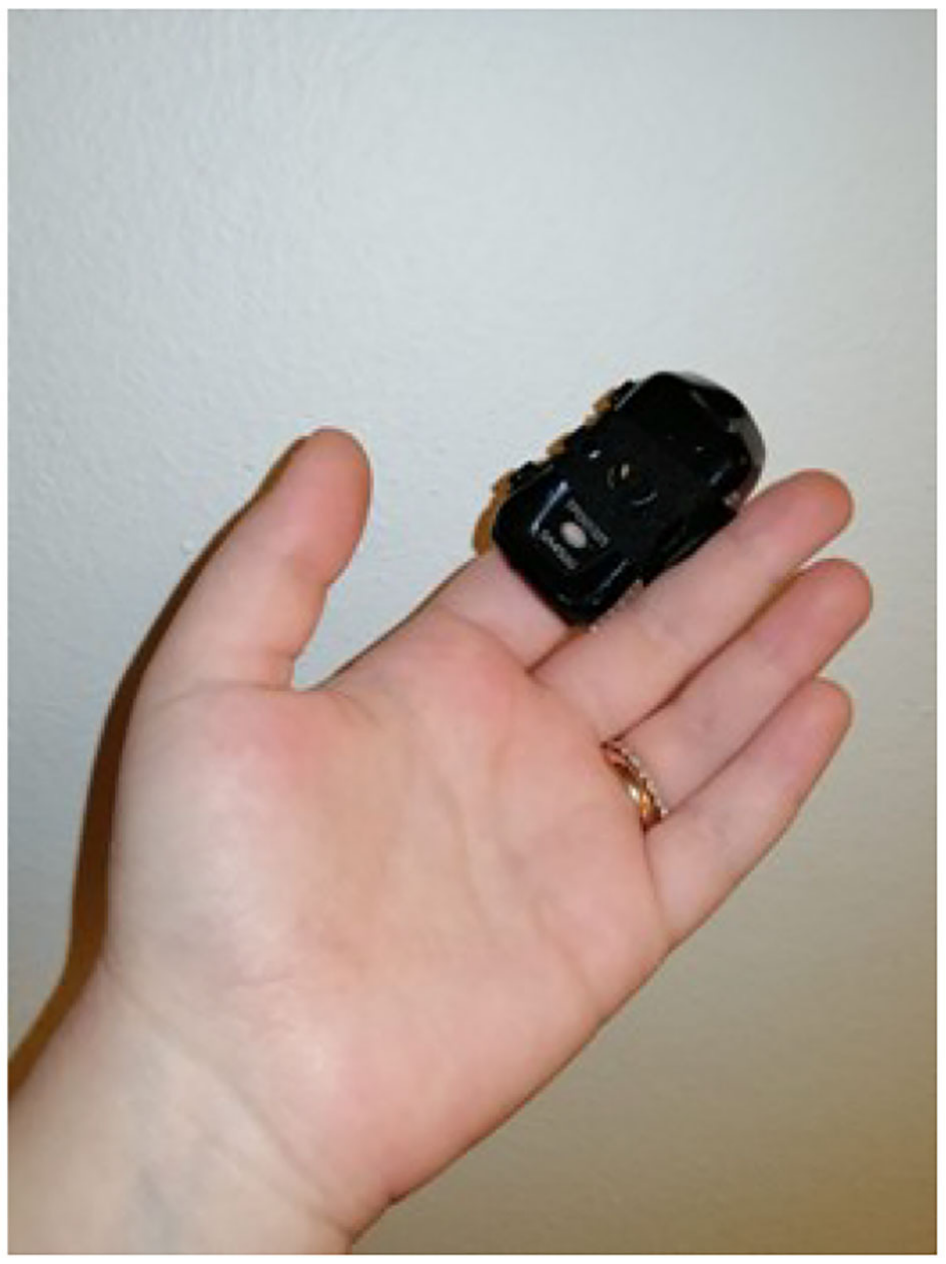
Evu Tps—biofeedback.

Software development included the creation of a virtual app, *Exludo*, which was interfaced with a computerized multiparametric acquisition system for biofeedback: EvU-TPS with BIOGRAPH INFINITI and DE-STRESS suite software (see Figure 10). This software includes the following features: video recording of the virtual sessions, psychophysiological parameter recording, and the integration of software that allows the therapist to configure the settings of the experience by operating in first person in the VR with a Drag'n'Drop interaction system. Drag and drop indicates a succession of three actions, consisting of clicking on a virtual object to drag it to another location within the virtual environment, where it is released. Virtual environments will remain the same for all patients and for the entire duration of the study. A wireless, cable-free kit was chosen for both the virtual hardware and biofeedback instrumentation for the patients' safety. Given the COVID-19 pandemic, appropriate accessories were also employed: disposable, non-woven, breathable face masks for HTC Vive PRO, waterproof and hygienic replaceable feather rubber for HTC Vive, and hygiene devices (spray, hand sanitizer gel, surgical masks) were used to guarantee the hygiene of the instruments and the patients' safety.

The design and implementation of the virtual environments that were created *ad hoc* for this study required a development period of about 6 months, in an interdisciplinary mode. Close collaboration between psychologists, programmers, and the 3D developers of Plumake S.r.l., who provided both the hardware and the *Exludo* software, was necessary. All of the environments were created *ex novo* and starting from real cues related to gambling (scratch cards, slot rooms, SuperEnalotto, snacks, cigarettes, etc.). The trade names of each of the 3D objects that were reproduced were changed due to the presence of the respective copyrights. After the creation of *Exludo*, a testing phase of the virtual hardware and software was planned and concluded in about 2 months. This phase was important in order to provide for the replacement of non-functional equipment and the correction of any bugs, i.e., errors in the writing of the software, which could interrupt the virtual experience or distort some of the environments' features. During the testing phase, a psychologist noticed some bugs in the movement system and in the interface between the biofeedback software and *Exludo*. Each environment was repeatedly tested to find as many errors as possible. The software has also been optimized in terms of usability, especially concerning the main start-up interface and the menu for selecting the actions to be taken. These changes guarantee an intuitive and immediate use of the application. A user guide was created by a psychologist and a programmer for the installation and the use of the hardware and software. A 10-h service package has been provided with respect to the hardware and software packages. Moreover, the correct functioning of the EvU-TPS biofeedback software had already been tested by the Medical Engineering Service of the Integrated University Hospital of Verona, and its interfacing with the virtual application was verified to ensure a reliable measurement of the parameters focused on the aims of *Alter Game* with respect to the detection of physiological activation due to craving. Righetto S.r.l. took care of the transport and assembly of the biofeedback instrumentation.

#### Measures

The second step of *Alter Game* was focused on the definition of the measures and procedure involved in the study.

##### Self-Report Instruments for the Assessment of Inclusion and Exclusion Criteria in the Intervention

Patients with GD between 18- and 65-years being part of the first and the second pathways of gamblers based upon Blaszczynski and Nower's classification (55) will be enrolled. They will comprise both” behaviorally conditioned problem gamblers” and” emotionally vulnerable problem gamblers.” The third pathway, comprising” antisocial impulsivist problem gamblers,” was excluded because these gamblers present high levels of impulsivity, and then they might be much more exposed to relapse after VR exposure than the other pathways.

The *Millon Clinical Multiaxial Inventory—III* [MCMI-III (56, 57)], the *Barratt Impulsiveness Rating Scale* [BIS-11 (58); Italian version (59)] and the *South Oaks Gambling Screen* [SOGS (60); Italian version (61)] will be used to evaluate the inclusion and exclusion criteria. The MCMI-III is a clinically oriented instrument, which is used in the assessment of personality disorders. The test comprises 175 dichotomous true/false items, 24 scales, and 4 correction indices. The questions investigate the presence of borderline and antisocial personality traits, thought disorders, and delusional disorders. The MCMI-III showed excellent construct validity, test-retest reliability, as well as internal consistency even if translated into several languages (62). Its factor structure is consistent across countries (63). The cut-off scores considered as exclusion criteria are: scores ≥ 75 on the “6A- Antisocial” scales in the “personality styles” section, “C-Borderline” in the “severe pathology” section, “SS-thinking disorders” and “PP-delusional disorders” in the “severe syndromes” section. Protocol invalidating scores: V scale: 2-3 points; X scale: ≤ 34 or ≥178.

The BIS-11 is a 30-item self-report scale, validated in Italian, which assesses impulsiveness. All items are rated on a 4-point scale, where 1 stands for rarely/never, and 4 stands for almost always/always. The BIS-11 proved to be a reliable psychometric instrument for measuring impulsiveness (59). The cut-off score considered as an exclusion criterion is > 65.46 + 12.08 (SD) (64).

The SOGS is a questionnaire that comprises 16 items that assess the presence of pathological gambling behavior. Good validity of the instrument has been demonstrated, particularly in terms of sensitivity and specificity (60), also in the Italian population (65). SOGS detects the type of gambling games played by gamblers. The cut-off score considered as an exclusion criterion is ≤ 4.

##### Pre and Post-test Follow-Up Measures

The *Gambling Related Cognitions Scale* [GRCS (66); Italian version (67)] and the *Multidimensional Gambling Self-Efficacy Scale* [MGSES (68)] will be used for the evaluation of changes in the experimental variables as a function of the treatment.

The GRCS is a 23-item scale that detects cognitive distortions on the basis of five specific erroneous thoughts: gambling expectations, illusion of control of fate, predictive control of an outcome, inability to stop gambling, and interpretative distortions. The patient responds to each item on a 7-point scale, ranging from 1 (completely disagree) to 7 (completely agree). Expectations related to gambling and the effects of gambling are an expression of the level of salience of the gambling experience and the effect expected from the activity of gambling. The illusion of control of fate is the belief that one can influence the outcome of the bet through the study and application of certain gambling strategies or by means of (magical) tools, such as lucky charms. Predictive control of the outcome is the prediction of the outcome of bets on the basis of feelings, perceptions of environmental traces that suggest the presence of positive or negative influences, or on the basis of pseudo-statistical reasoning on the probability of winning. The inability to stop gambling, i.e., the subjective feeling and belief of not being able to control the game is a dimension that expresses perceived self-efficacy. Interpretive distortions are thoughts that justify losses and encourage a person to return to gambling. The scale is characterized by good psychometric properties in terms of dimensionality, reliability, and validity both in its original version (66) and in the Italian adaptation (67).

The MGSES is a scale that was validated on the Italian population and comprises two sections: the first with 6 items and the second with 11 items, to be rated on a scale from 1 (not at all capable) to 5 (fully capable). The scale measures the gambler's perceived self-efficacy with respect to the management of gambling behavior and self-regulation during stressful situations, negative emotional states, free time, and other factors. The instrument showed good psychometric properties in terms of dimensionality, reliability, and validity in the Italian population (68).

##### Multidimensional Craving Measurement and Sense of Presence in VR

During VR therapy, craving and the sense of presence in VR training will be measured. Craving will be detected through both subjective and objective measures: a visual analog scale (VAS) to evaluate the perceived craving intensity on a scale from 0 to 10, based on the specific moment in which it will be administered. Furthermore, craving will be objectively evaluated with biofeedback EvU-TPS through the detection of the following psychophysiological measures: heart rate, heart rate variability, skin conductance, and skin temperature. The sense of presence will be measured through the ITC-SOPI self-report at the end of the virtual session. The Addiction Medicine Unit in collaboration with NEUROFARBA is working on a study to assess the psychometric properties of ITC-SOPI in its Italian version.

#### Training Course for Treatment Providers

After the development of the VR environments and the definition of the measures and procedures involved in the study, the next step consisted in training the treatment providers involved (psychologists and psychotherapists) and in a verification of learning after the training course. This represented a very important point as one of the factors that affect the efficacy of interventions in the health domain is the good quality of the training administered to the people who implement the programs (69). Indeed, training allows those who carry out the intervention to become confident with it (70). Moreover, some interventions need certain competencies that can be acquired only with specific training courses (71). In particular, a well-planned training course provides basic theoretical knowledge, clear program goals and objectives, modeling, and practice of effective intervention strategies, regular coaching, and constructive feedback (72). The training course consisted in 2 lessons on biofeedback, 2 lessons on CBT and diaphragmatic breathing, 1 lesson on VR theory, hardware and software application, and hygiene rules for virtual instruments. All lessons lasted 3 h, for a total duration of 15 h of training. Due to COVID-19 restrictions, the lessons were organized in a mixed modality, online and offline simultaneously. Two *ad hoc* questionnaires were created to assess the following variables: the first questionnaire had 13 questions and assessed electrodermal activity, skin conductance, photoplethysmography, heart rate, heart rate variability, the psychophysiological state of relaxation of the participants, their respiratory rate, and biofeedback training. The second questionnaire had ten questions and assessed respiratory training, mastery strategy, disputing, systematic desensitization, VR therapy, and cybersickness.

To analyze the efficacy of the training course for the psychotherapists involved in *Alter Game*, we proceeded to a pre-post design with a follow-up in which the above-listed variables were measured before the beginning of the training course (pre-test), after the last meeting of the training course (post-test), and 1 month after the conclusion of the course (follow-up). The treatment providers were provided with training material in the form of slides and guides to facilitate study and consultation in case of need. The coordinating psychologist sent the questionnaires *via* e-mail the day before the lessons for the pre-test, and then again after the end of each of the expected 2 training cycles. The first cycle included the 2 biofeedback lessons and, the second, the 3 lessons about CBT, diaphragmatic breathing, and VR. All training sessions were organized on the Meet platform, ensuring the mixed modality and the possibility to follow lessons in a deferred mode since everything was videotaped and made available to all the trainees on a shared drive folder. Training was provided by Righetto S.r.l. (as far as electromedical equipment was concerned) and by Plumake S.r.l. (for what concerned the hardware and virtual software dimension), respectively. The meetings concerning the training in psychotherapy techniques and VR therapy were organized by psychologist-psychotherapist collaborators.

#### Efficacy Evaluation of the Integrated Intervention

##### Design

We will conduct a pre-test/post-test experimental design with a 1-month follow-up.

Two independent groups to which patients will be randomly assigned will be considered. One group will undergo a traditional CBT treatment of 16 sessions (the CBT group), while the second group will undergo the integrated treatment, consisting of 8 CBT sessions and 8 sessions of psychotherapy based on VR cue-exposure therapy, each with a different environment (the CBT + VR group). Each session will last 1 h. To better control the effects of the training in VR on pathological gamblers, the psychophysiological parameters of craving will be measured through biofeedback and VAS at each session of the training (multi-method measurement of craving).

To verify the efficacy of the intervention, one measurement session is planned before the beginning of the treatment (pre-test), and one at the end of the treatment (post-test). For the assessment of the maintenance of any changes at post-test, an additional measurement is planned 1 month after the end of the treatment (follow-up). The three measurement sessions are planned for both the CBT group and the CBT + VR group. In each measurement session, perceived self-efficacy in managing gambling behavior, craving, gambling-related cognitive distortions, impulsivity, personality traits, and pathological gambling symptoms will be measured in both groups.

### Recruitment

We will recruit a sample of about 60 patients with GD who are referred to the Addiction Medicine Unit of Verona (G. B. Rossi Hospital). This sample size is consistent with the power analysis we conducted *a priori*. Using G^*^Power software to estimate the sample size that would be necessary to achieve a statistical power of 0.95 for a mixed ANOVA, indeed, revealed that a sample size of *n* = 58 was necessary in order to obtain an average effect size, equal to 0.40 (α = 0.05).

Inclusion criteria will be: (a) being male and female subjects over 18 years of age and under 65 years of age; (b) being addicted to slot machines, scratch cards, lotto, SuperEnalotto (because environments were created with these specific cues); (c) informed consent signature. An inclusion criterion (b) will be evaluated by a clinical interview based on the DSM-5 diagnostic criteria in the first psychological assessment session and by SOGS, which reports the type of gambling games played by gamblers.

Exclusion criteria will be the presence of: (a) antisocial personality traits; (b) borderline personality traits; (c) high levels of impulsivity; (d) thought disorders; (e) delusional disorders. Exclusion criteria (a), (b), and (c) were chosen to identify the presence of gamblers probably categorized as “third pathway” to Blaszczynski and Nower (55) and that, therefore, may be more exposed to risks of relapse following immersive VR exposure to environments with trigger cues. Excluding these patients will allow us to guarantee greater safety for them by administering treatment as usual.

We also identified particularly vulnerable populations that may not be included in the study: (a) patients suffering from epilepsy because HMD can provoke epileptic seizures like other devices that produce visual effects (as described in the HTC-VIVE official user's guide); (b) visually impaired patients and hearing impaired patients because they would have limitations in the use of the virtual experience concerning sounds and visual aspects; (c) patients with cardiovascular problems because our psychophysiological measures of interest could be altered by these pathologies; (d) gamblers with vestibular problems because they may fall during the virtual reality experience due to vertigo, motion sickness, and imbalance (73); (e) patients with neurological disorders: e.g., individuals with migraine are more susceptible to visual discomfort (74); (f) patients with schizophrenia or psychotic traits because the available data on immersive VR in schizophrenic subjects are currently limited (75), and no data were available on gamblers with psychotic diseases and immersive VR treatment.

Patients with the onset of cybersickness for two consecutive virtual sessions will exit from the study *in itinere* and will be able to undertake a path of psychotherapy as usual in the Addiction Medicine Unit.

### Interventional Methods

#### Intervention

Patients will be recruited when they access the Addiction Medicine specialist service “Colma il Gap” for GD at the G. B. Rossi Hospital in Verona, Italy. The selection of the sample will be based on the inclusion criteria. The selection of the patients who may be involved in the study will attend the following procedures. At the first access to the service, the patient will sign the informed consent and will be interviewed for the collection of anamnestic, life/family history, and clinical and gambling history details. We will assess legal and illegal substance use and the presence of addiction during the first psychological examination through a structured interview. It is important to capture sub-clinical factors related to the use of substances that could influence response to VR as well as situations linked with relapse. At the second visit, the questionnaires will be administered to the patient, in a self-completion mode, in the following order: MCMI-III, BIS-11, SOGS, GRCS, and MGSES. If the expected inclusion criteria will not be met, the patient will be excluded from the study but will be taken care of according to the usual treatment.

Patients meeting the inclusion criteria will be randomly assigned to the CBT group or to the CBT + VR group. The CBT group will follow a full cycle of psychotherapy that will consist of 16 weekly sessions, lasting 60 min and will make use of techniques belonging to an evidence-based cognitive-behavioral approach. For the CBT + VR group, there will be a first cycle of traditional psychotherapy that will consist of 8 weekly sessions, each lasting 60 min, which will also make use of evidence-based cognitive-behavioral approach techniques. Following this, psychotherapy based on virtual exposure will be carried out, which will again comprise 8 weekly sessions, marked by: systematic desensitization therapy in virtual reality exposure therapy, cognitive restructuring, and emotional management/literacy. Each virtual session will last 60 min, 20 min of which will be dedicated to virtual exposure. About 20 mins is the recommended limit of the duration of immersions in virtual reality because eyestrain and headache may occur for longer exposures (76, 77). We adopted this safety limit at least until empirical data document the safety of VR immersion with gamblers (10). An extinction process needs more time to originate, but limiting the duration of the immersion is necessary due to ethical considerations (10).

**Figure 11:**
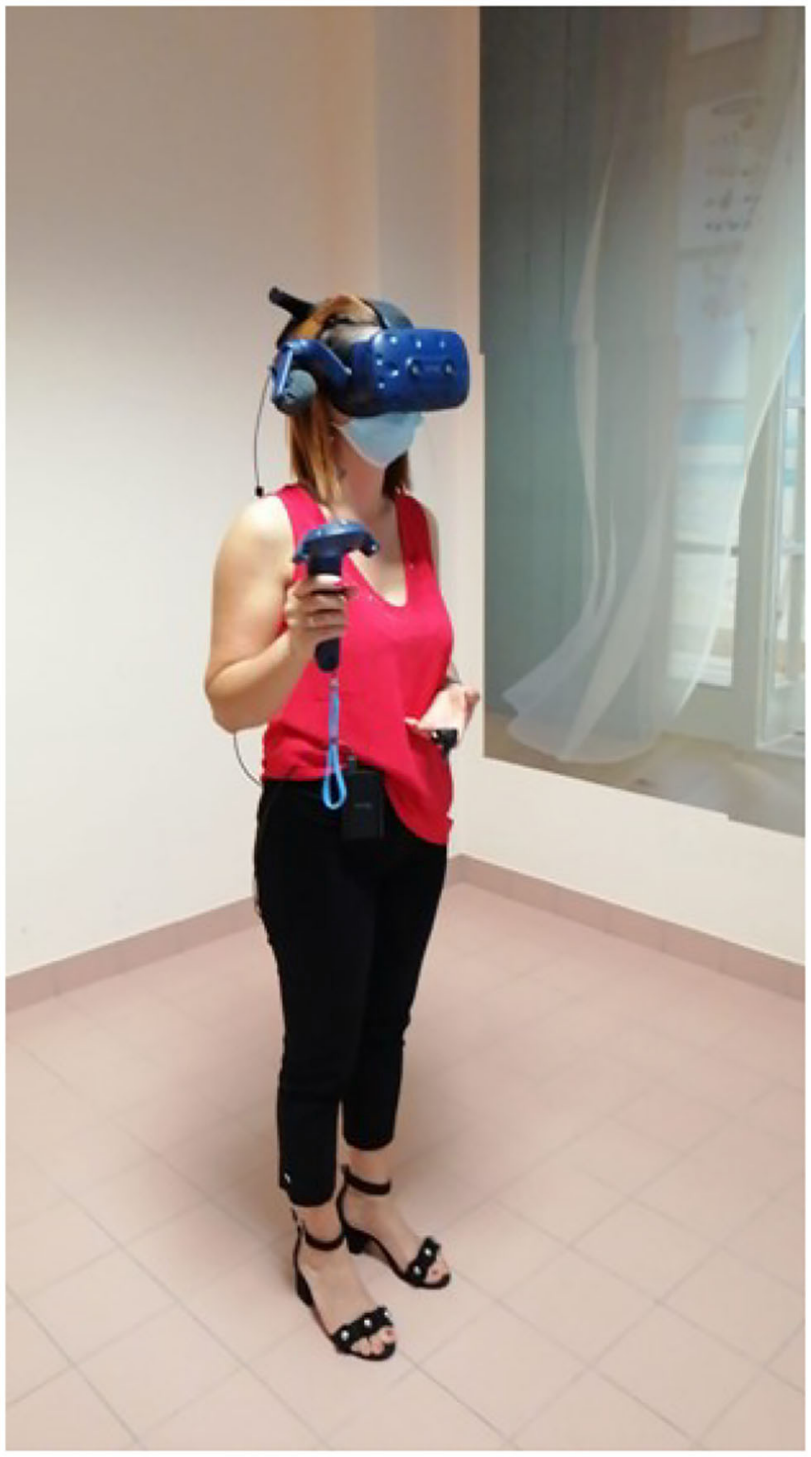
An example of a subject wearing the virtual reality and biofeedback equipment.

The virtual session will be structured as follows. In the first session, the virtual protocol will be presented to the patient, and virtual training will be carried out to provide the skills related to the use of the instrumentation (Figure 11). Biofeedback parameters will also be measured 5 min before the session. From the second to the eighth sessions, the biofeedback parameters will be measured 5 min before the beginning of the session (in the study, the baseline rate), and the measurements will be resumed during the entire exposure to the virtual environment. The patient will be asked about the intensity of the craving felt during both measurements and at the end of each session *via* VAS. As mentioned above, the technique used in conjunction with virtual cue exposure therapy will be systematic desensitization, which is a psychotherapeutic CBT treatment consisting in alternating phases of exposure to the trigger cues to phases of bodily relaxation, with the aim of reinforcing the relaxation response even in the presence of specific reactive cues (78, 79).

The systematic desensitization intervention will be structured as follows: the patient will learn a body relaxation technique [diaphragmatic breathing (80)]; the psychotherapist will proceed with the exposure of the patient to the trigger environments in the following order: a psychologist's office, a street with no game-related stimuli, a street with trigger stimuli, an empty tobacco shop, a full tobacco shop with sound stimuli, an empty slot room, a slot room with no audio-video stimuli, a slot room with active slot machines (audio-video stimuli), a slot room with active slot machines and alcohol and tobacco cues. During the virtual exposure to the trigger stimuli, when the patient signals that he/she is feeling a greater psychophysiological activation, the relaxation phase will begin, proceeding according to the following procedure: the patient will be teleported by the therapist to the psychologist's office (cue-free environment), where he/she will activate the radio to start the relaxing music and perform diaphragmatic breathing until psychophysical relaxation is achieved. When the patient is ready for the next exposure to the same trigger stimulus, the therapist will teleport him/her into the previous virtual trigger environment. The therapist will proceed in this way until the end of the time available for the virtual exposure. During the virtual exposure therapy, the psychotherapist will ask the patient to focus on bodily sensations and emerging thoughts and emotions related to gambling craving; after the 20 min of virtual exposure, the topics emerged will be the subject of psychotherapeutic work in the second part of the psychological interview. For both groups, the administration of the scales and questionnaires is scheduled for the nineteenth session; the results of which will be returned in the twentieth session. In the twenty-first session, the follow-up measurements will be carried out 1 month after the end of treatment to also verify the presence of any relapses. In detail, all the steps envisaged by *Alter Game* study are reported with the relative timing in the flow chart below (see Table 1); a total duration of about 18 months is expected (see Table 1).

**Table 1:** Flowchart of Alter Game study.

**Alter game steps**	**Timeline**							
	**Week 0**	**Week 1**	**Week 2**	**8 weeks**	**8 weeks**	**Week 19**	**Week 20**	**Week 25**
Design and implementation of virtual environments	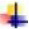							
Hardware and software installation and testing	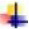							
Training course for Treatment Providers	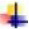							
First phone contact with patient	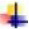							
Patient informed consent obtaining		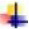						
Data collection about the patient demographic characteristics, medical history and clinical history concerning GD		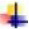						
Pre-test (MCMI-III, BIS-11, SOGS, GRCS, MGSES)			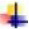					
Restitution of pre-test results to the patient				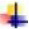				
Inclusion/exclusion criteria + randomization into CBT group and CBT + VR group				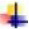				
First psychotherapy cycle (8 sessions of traditional psychotherapy for both groups)				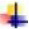				
Second psychotherapy cycle (8 sessions of traditional psychotherapy for CBT group and 8 sessions of psychotherapy based on virtual reality for CBT + VR group)					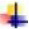			
Post-test (MCMI-III, BIS-11, SOGS, GRCS, MGSES)						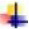		
Restitution of post-test results to the patient							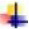	
1-month follow-up (MCMI-III, BIS-11, SOGS, GRCS, MGSES)								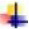

*GD, gambling disorder; MCMI-III, Millon Clinical Multiaxial Inventory-III; BIS-11, Barratt Impulsiveness Inventory-11; SOGS, South Oaks Gambling Screen; GRCR, Gambling Related Cognitions Scale; MGSES, Multidimensional Gambling Self-Efficacy Scale; CBT, cognitive behavioral therapy; VR, virtual reality*.

#### Safety and Hygiene Measures

To ensure patient safety and hygiene in the virtual setting, the recommendations of the Italian Higher Institute of Health (HIH) and the following procedures will be followed: (a) Hand cleaning: all subjects trained to handle VR devices shall have an alcohol-based hand sanitizer. Before a user turns on a device, he/she shall wash his/her hands for at least 40 s and following a precise sequence as illustrated on the Ministry of Health website; furthermore, he/she shall use a hydroalcoholic gel, rubbing his/her hands for 20 s (as described by HIH). (b) Ensuring that waterproof foam replacements are fitted to the HMD as the foam present by default does not allow for thorough hygiene. (c) Inserting disposable waterproof masks on HMD before using the viewer and replacing them between patients/treatment providers. (d) Disinfecting joysticks and all objects with which the patients and treatment providers may come into contact in between patients/treatment providers. (e) Sanitizing the EvU-TPS (biofeedback) instrumentation after each use. (f) Patients and treatment providers will be required to wear a surgical mask, respecting the indications of the HIH on the matter. (g) Ensuring that the patient has thoroughly cleaned his/her hands, both with soap and with sanitizing gel before entering the outpatient clinic; in fact, it will not be possible to make him/her wear gloves because of the need to measure psychophysiological parameters by photoplethysmography. (h) Repeating all hygiene procedures in between patients/treatment providers and at the end of each daily session so that the equipment remains sanitized at all times. (i) The hygiene of the surgery and the hospital environment will be guaranteed by the company staff dedicated to this purpose. (l) To sterilize the virtual reality devices, the product TRISEPT COMPLEX will be used, which is available at the warehouses of the internal pharmacy of the G.B. Rossi Hospital of Verona, Italy. (m) The use of an ultraviolet (UV) light box that sterilizes all virtual instrumentation without the use of chemicals that could damage the devices will be considered. Indeed, UV light can damage or destroy various types of viruses such as SARS and MERS (81). At the moment, some companies are working on some prototypes.

## Data Analysis

In general, all the patients' demographic and clinical characteristics will be summarized using descriptive statistical methods. In particular, mean, median, and standard deviations will be used for symmetrically distributed continuous variables, median and interquartile distance for asymmetrically distributed continuous variables, and frequency distributions for categorical variables. Multi-method analysis of craving measurement in order to draw up a physiological and psychometric profile of craving (using biofeedback) will be performed. This analysis will be conducted through linear correlation measures. For the analysis of the efficacy of the training course for the treatment providers, Student's *t*-tests for dependent samples or Wilcoxon's non-parametric test for paired samples will be used. Contingency tables and McNemar tests may also be used. For the analysis of the efficacy of the CBT + VR group compared to the CBT group, a mixed ANOVA will be performed for repeated measures with *Time* as within factor (pre-test/post-test/follow-up) and *Group* as between factor (the CBT + VR group/the CBT group).

Moreover, we will pay attention to the issue of an attrition rate. We are expecting to find a retention rate of at least 80% (78, 79) to avoid that participant loss will bias research outcomes. After that, we will check for any significant difference between the two treatment groups (CBT and CBT + VR) to verify if there is a differential attrition rate. In particular, we will compare the average number of meetings attended by the participants between the two treatment groups through *t*-tests, and we will compare the presence of drop-outs across the two groups through Chi-square tests. Moreover, we will determine if there are any significant differences (at the baseline) between participants who will complete the post-test session and those who will not in order to verify if specific subgroups of participants have a higher drop-out risk. This analysis will be conducted separately for each group. If any significant differences between participants of the two treatment groups will be found, we will verify that systematic attrition does not occur and we will conduct the analyses to verify the effects of the two interventions (CBT and CBT + VR) with the participants who completed the baseline and post-test assessments. Those lost to post tests will be excluded. If significant differences between participants of the two treatment groups will be found for the average number of meetings attended and the treatment drop-out, and participants who will complete the post-test session will be significantly different from those who will not, subsequent analyses will be conducted by using the last observation carried forward (LOCF) to assess the effects of attrition. Furthermore, we will examine within-group changes in outcomes as a function of session attendance to assess the effects of intervention non-compliance on outcomes. These subsequent analyses will be conducted, not excluding participants who will be lost to post-test and by imputing values for the follow-up variables for the participants who will not complete the post-test assessment.

## Discussion

A recent review has confirmed the efficacy of CBT to treat various addiction problems (7) and, especially, GD (9). Several published clinical studies have used VR to treat different psychopathological problems (anxiety, PTSD, depression, etc.), with a lot of them underlining that CBT and VR combined could improve the efficacy of social anxiety disorder and paranoia treatments (22, 24). Indeed, VR is a methodological approach that allows to recreate a realistic ecological representation of craving-eliciting situations (34–36). However, as of yet, no clinical study of CBT and VR in GD treatment has been conducted. Keeping this background into account, the necessity to develop a new study protocol—*Alter Game*– emerged. In detail, *Alter Game* aims at developing an innovative psychological treatment for GD by using VR and verifying the efficacy of an innovative psychological treatment for GD based on the integration of traditional CBT, coupled with a novel VR approach.

Based on previous studies, suggesting that CBT and VR combined are more efficient than CBT or VR only (23, 25), we aim at confirming this evidence in gambling addiction by taking specific target variables into account, such as self-efficacy, craving, and cognitive distortions. We can hypothesize that CBT and VR combined will be more effective than CBT only. In particular, at the conclusion of the psychotherapy sessions, we expect to find a higher increase of self-efficacy and a greater reduction of cravings and cognitive distortions—in terms of effect size—in the case of the integration of CBT and VR. To better evaluate these expected results, we aim at using biofeedback in order to observe and measure the psychophysiological activation associated with cue exposure, which would allow us to overcome the limits of self-report craving questionnaires or craving Likert scales. Moreover, we already implemented a training course for the treatment providers to guarantee a correct administration of the psychotherapy protocol with the patients before the study will start.

By increasing self-efficacy and reducing gambling-related craving and cognitive distortions, we aim at reducing the risk for relapse, which represents one of the most important psychotherapeutic steps for GD. Using VR cue-exposure therapy, pathological gamblers may increase their skills to prevent a relapse, or they could improve the management of their gambling craving and identify the triggers associated with it by pinpointing its cognitive, emotional, and bodily characteristics. These skills are important to prevent relapses in gambling behavior and to increase the self-efficacy of gamblers. By decreasing gambling-related distortions, which contribute to maintaining a maladaptive emotional regulation and reinforcing the irrational beliefs about gambling outcomes and controllability (48), we aim at promoting higher levels of management regarding thoughts about gambling in general. Another important aspect to be considered is that VR will provide contexts that are typical of everyday life, contributing to the generalizability of the craving management. Several studies using VR and cue reactivity to evaluate craving and other relapse risk factors in addictions (alcohol, tobacco, drugs, etc.) underlined the influence of life context in maintaining dependence or relapsing (82–84).

Despite these strengths, some limitations must be underlined. As a limitation of the study protocol, the use of self-report measures may be affected by the lack of insight/awareness, inability to identify or articulate beliefs/thoughts, and intentional dissimulation in pathological gamblers. However, the use of self-report measures is inevitable, so we chose tests that present good psychometrics properties, and we will consider the MCMI-III validity scales to control the social desirability index and the assessment protocol validity. In order to better control these biases, future studies could consider the introduction of behavioral tasks that in the Alter Game study protocol are not included except in the therapeutic intervention section. Also, the absence of a control group (non-treatment), which could serve to better analyze the efficacy of the integrated intervention, could be a further point to be addressed in the future. A public clinical service cannot omit to offer clinical treatment to a patient with GD for ethical reasons. Moreover, there may be potential threats to the internal validity of the research design due to its longitudinality, with a possible consequent loss of patients. In line with this, we chose a 1-month follow-up to keep the patients as engaged with the study as possible, since the dropout rate in pathological gamblers is high (18, 19). We are aware, however, that this time may not permit the detection of spontaneous recovery. Furthermore, studies could implement a strategy to better control susceptibility to conditioning. Another important consideration deals with the changes due to the COVID-19 pandemic. Indeed, GD is changing: a lot of patients are addicted to online gambling, so some patients that request our treatment may not respond to our inclusion criteria, in particular regarding the type of gambling activities considered in the *Alter Game* study before the beginning of the pandemic. In the future, the development and creation of new virtual environments on online gambling may be needed. The COVID-19 pandemic also brings additional complications: wearing the surgical mask can cause fogging problems to the HMD that compromise the correct use and the ideal fruition of the immersive virtual experience. In addition, the presence of restrictions for the containment of COVID-19 (lockdown, isolation, quarantines) may hamper the possibility of maintaining the 1 session per week that would be required by the study protocol. Another limitation regards the generalizability of the integrated protocol due to the possible presence of cybersickness and specific medical problems. Indeed, patients that present cybersickness during the virtual treatment or clinical problems like epilepsy, visual or hearing impairments, vestibular or cardiovascular or neurological diseases, and schizophrenia or psychotic traits will not be granted access to CBT + VR psychotherapy. Finally, future developments could improve our virtual environments through the insertion of avatars that interact with gamblers, thanks to artificial intelligence. This will be more expensive, but it may be useful to work on the impact of social interaction in the maintenance of GD and to manage situations in which gamblers are invited to eat snacks and drink alcoholic beverages. Indeed, it is known that social isolation and a sense of loneliness are related to problematic gambling (85, 86). Therefore, inserting social interaction in virtual gambling-related environments could be an important development to treat these daily experiences that may be relapse risk factors. Subjects that are socially excluded tend to self-medicate their unpleasant emotional states [e.g., anger, sadness, and anxiety; (87)] through emotion regulation processes (88) that can incorporate gambling too (89). Moreover, some studies highlighted that there is an association between gambling and alcohol use: those who drink alcohol more frequently are more inclined to gamble and to experience negative consequences due to their gambling behavior (90–93), as well as increased speed and duration of the game session (94, 95), increased risky wagering (94, 96, 97), and more rapid depletion of available funds (94–96).

To conclude, if our expected results will be confirmed, *Alter Game* could promote new possible tools for RP therapy that may be applicable within specialist-care services for GD and within therapeutic communities. These may also be suitable for poly-addicted gamblers, given the known high comorbidity between gambling and substance use disorders (98). Indeed, if the integrated virtual therapeutic protocol proposed by *Alter Game* will be effective, it could be extended to the treatment of other addiction disorders, reinforcing the traditional RP program.

## Ethics Statement

Approval for the research was obtained from the Ethics Committee for Clinical Trials (CESC) of the Provinces of Verona and Rovigo based at the Integrated University Hospital of Verona, Italy (approval code: 3004CESC with Protocol No. 69346 of 21/12/2020). The latest revision of the Declaration of Helsinki as well as the Oviedo Declaration is the basis for the ethical conduct of the study. The study protocol is designed and will be conducted to ensure adherence to the principles and procedures of Good Clinical Practice and to comply with Italian law, as described in the following documents and accepted, by signature, by the study investigators: ICH Harmonized Tripartite Guidelines for Good Clinical Practice 1996. Directive 91/507/EEC, The Rules Governing Medicinal Products in the European Community. D. L.vo n. 211 of June 24, 2003. D. L.vo n. 200, November 6 2007. Ministerial Decree of December 21, 2007. AIFA Determination, March 20, 2008. All essential clinical records will be retained to demonstrate the validity of the study and the integrity of the data collected. The promoter of this study, in accordance with the responsibilities required by the rules of good clinical practice (Legislative Decree 211/2003) and in accordance with the laws and regulations regarding data protection (including the European Regulation on the protection of personal data 2016/679), will process the personal data that will be collected exclusively for the implementation of the study and for the purpose of device surveillance.

## Author Contributions

FL and RG: conceptualization. RG: data curation and investigation. MD: statistical analysis. FL, RG, and MD: methodology. FL and MD: supervision. RG and LZ: writing—original draft. MD, FF, CP, and FL: writing—review and editing. All authors have read and agreed to the published version of the manuscript.

## Funding

The study described in this manuscript was funded by the Veneto Region (Italy) connected to the funding assigned by DGR n. 2154/2015, with the deliberation of the General Manager 1216 of 30/12/2016 to the Unit of Addiction Medicine in the Department of Internal Medicine in G.B. Rossi Hospital, Verona, Italy. The regional funding concerns the prevention and contrast of pathological gambling. The funding agency had no role in the design of the study or in writing this manuscript and will not influence the collection, analysis, and interpretation of data, apart from their financial contribution. All materials expected in the *Alter Game* study and all psychologists and psychotherapists involved were paid through Veneto Region's funding mentioned above.

## Conflict of Interest

The authors declare that the research was conducted in the absence of any commercial or financial relationships that could be construed as a potential conflict of interest.

## Publisher's Note

All claims expressed in this article are solely those of the authors and do not necessarily represent those of their affiliated organizations, or those of the publisher, the editors and the reviewers. Any product that may be evaluated in this article, or claim that may be made by its manufacturer, is not guaranteed or endorsed by the publisher.

## References

[1] American Psychiatric Association [APA]. Diagnostic and Statistical Manual of Mental Disorders, 5th edition. Arlington, VA: American Psychiatric Association (2013).

[2] Potenza MN. Should addictive disorders include non- substance-related conditions? Addiction. (2006) 101 (Suppl 1):142–51. 10.1111/j.1360-0443.2006.01591.x 16930171

[3] Weinstock J April LM Kallmi S. Is subclinical gambling really subclinical? Addict Behav. (2017) 73:185–91. 10.1016/j.addbeh.2017.05.014 28531824

[4] Slutske WS. Natural recovery and treatment-seeking in pathological gambling: results of two U.S. National surveys. Am J Psychiatry. (2006) 163:297–302. 10.1176/appi.ajp.163.2.297 16449485

[5] Slutske WS Blaszczynski A Martin NG. Sex differences in the rates of recovery, treatment-seeking, and natural recovery in pathological gambling: results from an Australian community-based twin survey. Twin Res Hum Genet. (2009) 12:425–32. 10.1375/twin.12.5.425 19803770

[6] Di Nicola M De Crescenzo F D'Alò GL Remondi C Panaccione I Moccia L . Pharmacological and psychosocial treatment of adults with gambling disorder: a meta-review. J Addict Med. (2020) 14:e15–23. 10.1097/ADM.0000000000000574 31651561

[7] Zamboni L Centoni F Fusina F Mantovani E Rubino F Lugoboni F . The effectiveness of cognitive behavioral therapy techniques for the treatment of substance use disorders: a narrative review of evidence. J NervMent Dis. (2021) 209:835–45. 10.1097/NMD.0000000000001381 34698698

[8] Bodor D Ricijaš N Filipčić I. Treatment of gambling disorder: review of evidence-based aspects for best practice. Curr Opin Psychiatry. (2021) 34:508–13. 10.1097/YCO.0000000000000728 34282103

[9] Cowlishaw S Merkouris S Dowling N Anderson C Jackson A Thomas S. Psychological therapies for pathological and problem gambling. Cochrane Database Syst Rev. (2012) 11:CD008937. 10.1002/14651858.CD008937.pub2 23152266 PMC11955261

[10] Giroux I Faucher-Gravel A St-Hilaire A Boudreault C Jacques C Bouchard S. Gambling exposure in virtual reality and modification of urge to gamble. Cyberpsychol Behav Soc Netw. (2013) 16:224–31. 10.1089/cyber.2012.1573 23496679

[11] Rizeanu S. Proposal for a cognitive model to the treatment of pathological gambling. ProcediaSocBehavSci. (2012) 33:742–6. 10.1016/j.sbspro.2012.01.220

[12] Marlatt GA Gordon JR editors. Relapse Prevention: Maintenance Strategies in the Treatment of Addictive Behaviors. New York: Guilford Press (1985).

[13] Larimer ME Palmer RS Marlatt GA. Relapse prevention: an overview of Marlatt's cognitive-behavioral model. Alcohol Res Health. (1999) 23:151–60. 10890810 PMC6760427

[14] Bandura A. Self-efficacy: toward a unifying theory of behavioral change. Psychol Rev. (1977) 84:191–215. 10.1037/0033-295X.84.2.191 847061

[15] Symes BA Nicki RM. A preliminary consideration of cue exposure, response-prevention treatment for pathological gambling behaviour: two case studies. J Gambl Stud. (1997) 13:145–57. 10.1023/A:1024951301959 12913392

[16] Raylu N Oei TP. A Cognitive Behavioural Therapy Programme for Problem Gambling. Routledge: East Sussex (2010).

[17] Rizeanu S. Psychological profile of the Romanian pathological gambler. ProcediaSocBehavSci. (2014) 127:265–9. 10.1016/j.sbspro.2014.03.253

[18] Jiménez-Murcia S Granero R Fernández-Aranda F Arcelus J Aymamí MN Gómez-Peña M . Predictors of out come among pathological gamblers receiving cognitive behavioral group therapy. Eur Addict Res. (2015) 21:169–78. 10.1159/000369528 25832435

[19] Tárrega S Castro-Carreras L Fernández-Aranda F Granero R Giner-Bartolomé C Aymamí N . A serious videogame as an additional therapy tool for training emotional regulation and impulsivity control in severe gambling disorder. Front Psychol. (2015) 6:1721. 10.3389/fpsyg.2015.01721 26617550 PMC4641919

[20] Firth J Torous J Nicholas J Carney R Pratap A Rosenbaum S . The efficacy of smartphone-based mental health interventions for depressive symptoms: a meta-analysis of randomized controlled trials. World Psychiatry. (2017) 16:287–98. 10.1002/wps.20472 28941113 PMC5608852

[21] Wiederhold BK Bouchard S. Advances in Virtual Reality and Anxiety Disorders. New York: Springer US (2014).

[22] Rizzo AA Graap K Perlman K McLay RN Rothbaum BO Reger G . Virtual Iraq: initial results from a VR exposure therapy application for combat-related PTSD. Stud Health Technol Inform. (2008) 132:420–5. 10.1109/ICVR.2007.4362152 18391334

[23] Pelissolo A AbouKassm S Delhay L. Therapeutic strategies for social anxiety disorder: where are we now? Expert Rev Neurother. (2019) 19:1179–89. 10.1080/14737175.2019.1666713 31502896

[24] Morina N Brinkman WP Hartanto D Kampmann IL Emmelkamp PM. Social interactions in virtual reality exposure therapy: a proof-of-concept pilot study. Technol Health Care. (2015) 23:581–9. 10.3233/THC-151014 26410119

[25] Geraets CNW Snippe E van Beilen M Pot-Kolder RMCA Wichers M van der Gaag M . Virtual reality based cognitive behavioral therapy for paranoia: effects on mental states and the dynamics among them. Schizophr Res. (2020) 222:227–34. 10.1016/j.schres.2020.05.047 32527676

[26] Rothbaum BO Hodges LF Kooper R Opdyke D Williford JS North M. Effectiveness of computer-generated (virtual reality) graded exposure in the treatment of acrophobia. Am J Psychiatry. (1995) 152:626–8. 10.1176/ajp.152.4.626 7694917

[27] Rothbaum BO Hodges L Smith S Lee JH Price L. A controlled study of virtual reality exposure therapy for the fear of flying. J Consult Clin Psychol. (2000) 68:1020–6. 10.1037/0022-006X.68.6.1020 11142535

[28] Rothbaum BO Hodges L Anderson PL Price L Smith S. Twelve-month follow-up of virtual reality and standard exposure therapies for the fear of flying. J Consul Clin Psychol. (2002) 70:428–32. 10.1037/0022-006X.70.2.428 11952201

[29] Bouchard S Côté S St-Jacques J Robillard G Renaud P. Effectiveness of virtual reality exposure in the treatment of arachnophobia using 3D games. Technol Health Care. (2006) 14:19–27. 10.3233/THC-2006-14103 16556961

[30] Tardif N Therrien CÉ Bouchard S. Re-examining psychological mechanisms underlying virtual reality-based exposure for spider phobia. Cyberpsychol Behav Social Netw. (2019) 22:39–45. 10.1089/cyber.2017.0711 30256675

[31] Bouchard S Robillard G Giroux I Jacques C Loranger C St-Pierre M . Using virtual reality in the treatment of gambling disorder: the development of a new tool for cognitive behavior therapy. Front Psychiatry. (2017) 8:27. 10.3389/fpsyt.2017.00027 28286486 PMC5324022

[32] Park CB Park SM Gwak AR Sohn BK Lee JY . The effect of repeated exposure to virtual gambling cues on the urge to gamble. Addict Behav. (2015) 41:61–4. 10.1016/j.addbeh.2014.09.027 25306387

[33] Garcia-Palacios A Lasso de la Vega N Botella C Banos R Quero S. Virtual reality in the treatment of pathological gambling. In: Oral Presentation at the 11th Annual Cybererapy 2006 Conference; June 13–15. Gatineau, Canada.

[34] Kushner MG Abrams K Donahue C Thuras P Frost R Kim SW. Urge to gamble in problem gamblers exposed to a casino environment. J Gambling Studies. (2007) 23:121–32. 10.1007/s10899-006-9050-4 17245663

[35] Thewissen R Van Den Hout M Havermans RC Jansen A. Context-dependency of cueelicited urge to smoke. Addiction. (2005) 100:387–96. 10.1111/j.1360-0443.2005.00996.x 15733252

[36] Conklin CA Robin N Perkins KA Salkeld RP McClernon FJ. Proximal versus distal cues to smoke: the effects of environments on smokers' cue-reactivity. Hum PsychopharmClin. (2008) 16:207–14. 10.1037/1064-1297.16.3.207 18540780 PMC2963185

[37] Traylor AC Parrish DE Copp HL Bordnick TS. Using virtual reality to investigate complex and contextual cue reactivity in nicotine dependent problem drinkers. Addict Behav. (2011) 36, 1068–1075. 10.1016/j.addbeh.2011.06.014 21783326

[38] Martinotti G Di Nicola M Tedeschi D Callea A Di Giannantonio M Janiri L Craving Study Group. Craving Typology Questionnaire (CTQ): a scale for alcohol craving in normal controls and alcoholics. Comprehens Psychiatry. (2013) 54:925–32. 10.1016/j.comppsych.2013.03.023 23642635

[39] Andrade C. Internal, external, and ecological validity in research design, conduct, and evaluation. Indian J Psychol Med. (2018) 40:498–9. 10.4103/IJPSYM.IJPSYM_334_18 30275631 PMC6149308

[40] Gallagher M Ferrè ER. Cybersickness: a multisensory integration perspective. Multisensory Res. (2018) 31:645–74. 10.1163/22134808-20181293 31264611

[41] Rebenitsch L Owen C. Review on cybersickness in applications and visual displays. Virtual Reality. (2016) 20:101–25. 10.1007/s10055-016-0285-934276550

[42] Sheridan TB. Musings on telepresence and virtual presence. Presence Teleoper Virtual Environ. (1992) 1:120–6. 10.1162/pres.1992.1.1.120

[43] Barfield W Zeltzer D Sheridan TB Slater M. Presence and performance within virtual environments. In: Barfield W, Furness TA, editors. Virtual Environments and Advanced Interface Design (pp. 473–541). Oxford: Oxford University Press (1995).

[44] Lessiter J Freeman J Keogh E Davidoff J. A cross-media presence questionnaire: the ITC-sense of presence inventory. Presence Teleoper Virtual Environ. (2001) 10:282–97. 10.1162/105474601300343612

[45] Carter BL Tiffany ST. Meta-analysis of cue-reactivity in addiction research. Addiction. (1999) 94:327–40. 10.1046/j.1360-0443.1999.9433273.x 10605857

[46] Ashrafioun L Rosenberg H. Methods of assessing craving to gamble: a narrative review. Psychol Addict Behavs. (2012) 26:536–49. 10.1037/a0026367 22121917

[47] Schluter MG Kim HS Poole JC Hodgins DC McGrath DS Dobson KS . Gambling-related cognitive distortions mediate the relationship between depression and disordered gambling severity. Addict Behav. (2019) 90:318–23. 10.1016/j.addbeh.2018.11.038 30503951

[48] Ruiz de Lara CM Navas JF Perales JC. The paradoxical relationship between emotion regulation and gambling-related cognitive biases. PloS ONE. (2019) 14:e0220668. 10.1371/journal.pone.0220668 31381598 PMC6681951

[49] López-Torres I León-Quismondo L Ibáñez A. Impulsivity, lack of premeditation, and debts in online gambling disorder. Front Psychiatry. (2021) 11:618148. 10.3389/fpsyt.2020.618148 33551878 PMC7855030

[50] Brosowski T Olason DT Turowski T Hayer T. The gambling consumption mediation model (GCMM): a multiple mediation approach to estimate the association of particular game types with problem gambling. J Gambling Stud. (2021) 37:107–40. 10.1007/s10899-020-09928-3 31965383 PMC7882568

[51] Delfabbro P Parke J. Empirical evidence relating to the relative riskiness of scratch-card gambling. J Gambling Stud. (2021) 37:1007–24. 10.1007/s10899-021-10033-2 33969455

[52] Barrus MM Cherkasova M Winstanley CA. Skewed by cues? The motivational role of audiovisual stimuli in modelling substance use and gambling disorders. Curr Topics Behav Neurosci. (2016) 27:507–29. 10.1007/7854_2015_393 26531068

[53] Griffiths M. Fruit machine gambling: the importance of structural characteristics. J Gambling Stud. (1993) 9:101–120. 10.1007/BF01014863

[54] Weech S Kenny S Barnett-Cowan M. Presence and cybersickness in virtual reality are negatively related: a review. Front Psychol. (2019) 10:158. 10.3389/fpsyg.2019.00158 30778320 PMC6369189

[55] Blaszczynski A Nower L. A pathways model of problem and pathological gambling. Addiction. (2002) 97:487–99. 10.1046/j.1360-0443.2002.00015.x 12033650

[56] Millon T. MCMI- III Manual, 3rd Edition. Minneapolis, MN: NCS Pearson (2006). p. 16

[57] Millon T. MCMI III, MillonClinicalMultiaxial Inventory Iii Ed., Manuale. (2008). Trad. It. a Cura di: Zennaro A, Ferracuti S, Lang M, Sanavio e Eds. Firenze: GiuntiOs.

[58] Patton JH Stanford MS Barratt ES. Factor structure of the Barratt impulsiveness scale. J Clin Psychol. (1995) 51:768–74. 10.1002/1097-4679(199511)51:6<768::AID-JCLP2270510607>3.0.CO;2-1 8778124

[59] Fossati A Di Ceglie A Acquarini E Barratt ES. Psychometric properties of an Italian version of the Barratt Impulsiveness Scale-11 (BIS-11) in nonclinical subjects. J Clin Psychol. (2001) 57:815–28. 10.1002/jclp.1051 11344467

[60] Lesieur HR Blume SB. The South Oaks Gambling Screen (SOGS): a new instrument for the identification of pathological gamblers. Am J Psychiatry. (1987) 144:1184–8. 10.1176/ajp.144.9.1184 3631315

[61] Guerreschi C Gander S. Versione Italiana del South Oaks Gambling Screen (SOGS) di H.R. Lesieur e S.B. Blume. In: C. Guerreschi, editor. Giocati dal gioco. Quando il divertimento diventa una malattia: il gioco d'azzardo patologico. San Paolo: Milano (2000), p. 137–42.

[62] Alareqe NA Roslan S Nordin MS Ahmad NA Taresh SM. Psychometric Properties of the Millon Clinical Multiaxial Inventory-III in an Arabic clinical sample compared with American, Italian, and Dutch Cultures. Front Psychol. (2021) 12:562619. 10.3389/fpsyg.2021.562619 34566736 PMC8458952

[63] Pignolo C Rosalo R Andò A Cristofanelli S Ferro L Zennaro A. The factor structure of the Italian version of the MCMI-III compared to the Dutch and American versions. BPA Appl Psychol Bull. (2017) 65:36–46.

[64] Marazziti D Picchetti M Baroni S Consoli G Ceresoli D Massimetti G . Pathological gambling and impulsivity: an Italian study. Riv Psichiatria. (2014) 49:95–9. 10.1708/1461.16149 24770575

[65] Barbaranelli C Vecchione M Fida R Podio-Guidugli S. Estimating the prevalence of adult problem gambling in Italy with SOGS and PGSI. J Gambling Issues. (2013) 28:1–24. 10.4309/jgi.2013.28.3

[66] Raylu N Oei TP. The Gambling Related Cognitions Scale (GRCS): development, confirmatory factor validation and psychometric properties. Addiction. (2004) 99:757–69. 10.1111/j.1360-0443.2004.00753.x 15139874

[67] Iliceto P Fino E Cammarota C Giovani E Petrucci F Desimoni M . Factor structure and psychometric properties of the Italian version of the Gambling Related Cognitions Scale (GRCS-I). J Gambling Stud. (2015) 31:225–42. 10.1007/s10899-013-9405-6 23949626

[68] Barbaranelli C Ghezzi V Fida R Vecchione M. Psychometric characteristics of a new scale for measuring self-efficacy in the regulation of gambling behavior. Front Psychol. (2017) 8:1025. 10.3389/fpsyg.2017.01025 28676781 PMC5477641

[69] Nation M Crusto C Wandersman A Kumpfer KL Seybolt D Morrissey-Kane E . What works in prevention: principles of effective prevention programs. Am Psychol. (2003) 58:449–56. 10.1037/0003-066X.58.6-7.449 12971191

[70] Ahmed N Flisher AJ Mathews C Jansen S Mukoma W Schaalma H. Process evaluation of the teacher training for an AIDS prevention programme. Health Educ Res. (2006) 21:621–32. 10.1093/her/cyl031 16740671

[71] Schaalma HP Abraham C Gillmore MR Kok G. Sex education as health promotion: what does it take? Arch Sex Behav. (2004) 33:259–69. 10.1023/B:ASEB.0000026625.65171.1d 15129044

[72] Hall G Hord S. Implementing Change: Patterns, Principles and Potholes. Boston, MA: Allyn and Bacon (2001).

[73] Viirre E. Vestibular telemedicine and rehabilitation. Applications for virtual reality. Stud Health Technol Inform. (1996) 29:299–305. 10163763

[74] O'Hare L Sharp A Dickinson P Richardson G Shearer J. Investigating head movements induced by 'Riloid' patterns in migraine and control groups using a virtual reality display. Multisens Res. (2018) 31:753–77. 10.1163/22134808-20181310 31264621

[75] Bisso E Signorelli MS Milazzo M Maglia M Polosa R Aguglia E . Immersive Virtual reality applications in schizophrenia spectrum therapy: a systematic review. Int J Environ Res Public Health. (2020) 17:6111. 10.3390/ijerph17176111 32842579 PMC7504018

[76] Bouchard S Cote' S Richard DS. Virtual reality applications of exposure. In: Richard DS, Lauterbach D, editors. Handbook of Exposure (Ch. 16). New York: Academic Press (2006). p. 347–88.

[77] Bouchard S St-Jacques J Renaud P Wiederhold BK. Side effects of immersion in Virtual Reality for people suffering from anxiety disorders. J Cybertherapy Rehabil. (2009) 2:127–37. 10.3233/SHTI210961 35062194

[78] Fischer EH Dornelas EA Goethe JW. Characteristics of people lost to attrition in psychiatric follow-up studies. J Nervous Mental Dis. (2006) 189:49–55. 10.1097/00005053-200101000-00009 11206665

[79] Wilkins W. Desensitization: social and cognitive factors underlying the effectiveness of Wolpe's procedure. Psychol Bull. (2001) 76:311–7. 4942080 10.1037/h0031722

[80] Russell ME Scott AB Boggero IA Carlson CR. Inclusion of a rest period in diaphragmatic breathing increases high frequency heart rate variability: implications for behavioral therapy. Psychophysiology. (2017) 54:358–65. 10.1111/psyp.12791 27925652 PMC5319881

[81] Türsen Ü Türsen B Lotti T. Ultraviolet and COVID-19 pandemic. J Cosmet Dermatol. (2020) 19:2162–4. 10.1111/jocd.13559 32573919 PMC7361848

[82] Gilpin EA Messer K Pierce JP. Population effectiveness of pharmaceutical aids for smoking cessation: what is associated with increased success? Nicotine Tob Res. (2006) 8:661–9. 10.1080/14622200600910801 17008193

[83] Pericot-Valverde I Germeroth LJ Tiffany ST. The use of virtual reality in the production of cue-specific craving for cigarettes: a meta-analysis. Nicotine Tob Res. (2015) 18:538–46. 10.1093/ntr/ntv216 26453669

[84] Chiamulera C Ferrandi E Benvegnù G Ferraro S Tommasi F Maris B . Virtual reality for neuroarchitecture: cue reactivity in built spaces. Front Psychol. (2017) 8:185. 10.3389/fpsyg.2017.00185 28243216 PMC5303754

[85] Castrén S Basnet S Salonen AH Pankakoski M Ronkainen J-E Alho H . Factors associated with disordered gambling in Finland. Subst Abuse Treat Prev Policy. (2013) 8:24. 10.1186/1747-597X-8-24 23816162 PMC3706208

[86] McQuade A Gill P. The role of loneliness and self-control in predicting problem gambling behaviour. Gambling Res. (2012) 24:18–30.

[87] Buckley KE Winkel RE Leary MR. Reactions to acceptance and rejection: effects of level and sequence of relational evaluation. J Exp Social Psychol. (2004) 40:14–28. 10.1016/S0022-1031(03)00064-7

[88] Riva P. Emotion regulation following social exclusion: psychological and behavioral strategies. In: Riva P, Eck J, editor. Social Exclusion: Psychological Approaches to Understanding and Reducing its Impact. New York, NY: Springer (2016). p. 199–226.

[89] Pancani L Riva P Sacchi S. Connecting with a slot machine: social exclusion and anthropomorphization increase gambling. J Gambl Stud. (2019) 35:689–707. 10.1007/s10899-018-9784-9 29959690

[90] Welte J Barnes G Wieczorek W Tidwell MC Parker J. Alcohol and gambling pathology among U.S. adults: prevalence, demographic patterns and comorbidity. J Stud Alcohol. (2001) 62:706–12. 10.15288/jsa.2001.62.706 11702810

[91] Blankenship J Starling R Woodall WG May PA. Gambling and alcohol use: trends in the state of New Mexico from 1996–1998. J Gambling Stud. (2007) 23:157–74. 10.1007/s10899-006-9051-3 17318399

[92] French MT Maclean JC Ettner SL. Drinkers and bettors: investigating the complementarity of alcohol consumption and problem gambling. Drug Alcohol Depend. (2008) 96:155–64. 10.1016/j.drugalcdep.2008.02.011 18430523 PMC2710110

[93] Huggett SB Winiger EA Corley RP Hewitt JK Stallings MC. Alcohol use, psychiatric disorders and gambling behaviors: a multi-sample study testing causal relationships via the co-twin control design. Addict Behav. (2019) 93:173–9. 10.1016/j.addbeh.2019.01.024 30716592 PMC7170180

[94] Ellery M Stewart SH Loba P. Alcohol's effects on video lottery terminal (VLT) play among probable pathological and non-pathological gamblers. J Gambling Stud. (2005) 21:299–324. 10.1007/s10899-005-3101-0 16134010

[95] Phillips JG Ogeil RP. Alcohol consumption and computer blackjack. J Gen Psychol. (2007) 134:333–53. 10.3200/GENP.134.3.333-354 17824402

[96] Cronce JM Corbin WR. Effects of alcohol and initial gambling outcomes on within-session gambling behavior. Exp Clin Psychopharmacol. (2010) 18:145–57. 10.1037/a0019114 20384426 PMC3039524

[97] Ellery M Stewart SH. Alcohol affects video lottery terminal (VLT) gambling behaviors and cognitions differently. Psychol Addict Behav. (2014) 28:206–16. 10.1037/a0035235 24731116

[98] Hynes TJ Hrelja KM Hathaway BA Hounjet CD Chernoff CS Ebsary SA . Dopamine neurons gate the intersection of cocaine use, decision making, and impulsivity. Addictionbiology. (2021) 26:e13022. 10.1111/adb.13022 33559379

